# On‐Surface Synthesis of Cumulene‐Containing Polymers via Two‐Step Dehalogenative Homocoupling of Dibromomethylene‐Functionalized Tribenzoazulene

**DOI:** 10.1002/anie.202001939

**Published:** 2020-05-29

**Authors:** José I. Urgel, Marco Di Giovannantonio, Kristjan Eimre, Thorsten G. Lohr, Junzhi Liu, Shantanu Mishra, Qiang Sun, Amogh Kinikar, Roland Widmer, Samuel Stolz, Max Bommert, Reinhard Berger, Pascal Ruffieux, Carlo A. Pignedoli, Klaus Müllen, Xinliang Feng, Roman Fasel

**Affiliations:** ^1^ Empa – Swiss Federal Laboratories for Materials Science and Technology Überlandstrasse 129 8600 Dübendorf Switzerland; ^2^ Center for Advancing Electronics and Department of Chemistry and Food Chemistry Technical University of Dresden 01062 Dresden Germany; ^3^ Laboratory of Nanostructures at Surfaces Institute of Physics, École Polytechnique Fédérale de Lausanne CH-1015 Lausanne Switzerland; ^4^ Max Planck Institute for Polymer Research Ackermannweg 10 55128 Mainz Germany; ^5^ Department of Chemistry and Biochemistry University of Bern Freiestrasse 3 3012 Bern Switzerland

**Keywords:** carbon nanostructures, cumulenes, nonbenzenoid hydrocarbons, sequential dehalogenation, surface chemistry

## Abstract

Cumulene compounds are notoriously difficult to prepare and study because their reactivity increases dramatically with the increasing number of consecutive double bonds. In this respect, the emerging field of on‐surface synthesis provides exceptional opportunities because it relies on reactions on clean metal substrates under well‐controlled ultrahigh‐vacuum conditions. Here we report the on‐surface synthesis of a polymer linked by cumulene‐like bonds on a Au(111) surface via sequential thermally activated dehalogenative C−C coupling of a tribenzoazulene precursor equipped with two dibromomethylene groups. The structure and electronic properties of the resulting polymer with cumulene‐like pentagon–pentagon and heptagon–heptagon connections have been investigated by means of scanning probe microscopy and spectroscopy methods and X‐ray photoelectron spectroscopy, complemented by density functional theory calculations. Our results provide perspectives for the on‐surface synthesis of cumulene‐containing compounds, as well as protocols relevant to the stepwise fabrication of carbon–carbon bonds on surfaces.

## Graphical abstract

**Superficial interactions**: On‐surface investigations of tribenzoazulene molecules functionalized with two dibromomethylene groups at the pentagon and heptagon ends reveal, after sequential annealing steps, the unambiguous formation of polymers linked by cumulene‐like bonds via selective C−C coupling induced by the sequential activation of the precursor molecules on a Au(111) surface.

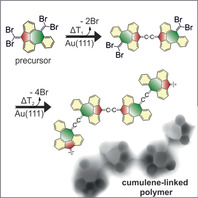

## Introduction

Over the last century, the well‐known carbon allotropes with sp^2^‐ (graphite) and sp^3^‐ (diamond) hybridization have been extensively investigated due to their relevance, ranging from drugs to synthetic materials of interest in many applications because of their high surface area and physicochemical properties.^1^, ^2^, ^3^ Only in 1985, fullerenes were observed for the first time,^4^ opening a new era for synthetic carbon allotropes. Since then, many more allotropes such as graphene,^5^ carbon nanotubes,^6^ nanobuds,^7^ and schwarzites^8^ have been discovered, bringing revolutionary developments in diverse fields ranging from nanoelectronics to catalysis and energy devices. Nevertheless, much less is known about carbon allotropes with sp‐hybridization. Carbyne, a linear chain of sp‐hybridized carbon atoms,^9^ has been the subject of controversy over the last decades.^10^ It can exist in two different forms: either as polyyne, where the carbon atoms that compose the linear chain are linked by alternate single and triple bonds (‐C≡C‐), or as cumulene with carbon atoms linked by consecutive double bonds (=C=C=).^11^ While the synthesis of polyynes has been widely explored,^12^, ^13^, ^14^ investigations on cumulene compounds have remained scarce as a result of the drastic increase in reactivity as the number of consecutive cumulene bonds increases.^15^, ^16^, ^17^

Recently, traditional solution synthesis has been extended to the study of chemical reactions on single‐crystal surfaces under ultrahigh‐vacuum (UHV) conditions, which provides a versatile playground for the investigation of novel surface‐supported nanostructures that are difficult to achieve in solution. The use of halogen leaving groups at specific sites of the molecules (with low bond energies in comparison to the molecular backbone) has emerged as a particularly successful approach in the study of on‐surface reactions.^15^ On metal substrates, moderate heating readily induces dehalogenation and thus reactive radical sites, which, together with surface diffusion of the activated molecules, leads to the desired formation of covalent (carbon–carbon) bonds. Nevertheless, a stepwise pathway to covalent linking reactions on surfaces has been achieved only rarely.^16^ Over the last decades, a plethora of on‐surface reactions based on C−C coupling such as (dehalogenative) aryl–aryl coupling,^8^, ^15^, ^17^, ^18^ and cyclodehydrogenation^19^ have been studied via scanning probe microscopy (SPM) and other surface analysis techniques. Only recently, the formation of cumulene‐containing dimers and polymers by dehalogenative homocoupling reactions of gem‐dibromides^20^, ^21^ and by enediyne coupling,^22^ together with the generation of several intermediates on different surfaces via scanning tunneling microscopy (STM)‐based manipulation has been reported.^23^, ^24^ However, detailed studies of the on‐surface synthesis of cumulene‐containing compounds and their in‐depth structural and electronic characterization have remained elusive. Here, we report on a comprehensive STM, scanning tunneling spectroscopy (STS), non‐contact atomic force microscopy (nc‐AFM), X‐ray photoelectron spectroscopy (XPS), and density functional theory (DFT) study of the surface‐confined formation of a one‐dimensional polymer linked by cumulene‐like bonds. Importantly, the specific adsorption geometry of the molecular precursor 1,5‐bis(dibromomethylene)‐1,5‐dihydrobenzo[5,6]cyclohepta[1,2,3,4‐*def*]fluorene (**1**) on Au(111) leads to two coupling steps separated in temperature that result in a selective head‐to‐head/tail‐to‐tail monomer sequence in the polymer.

## Results and Discussion

**Scheme 1:**
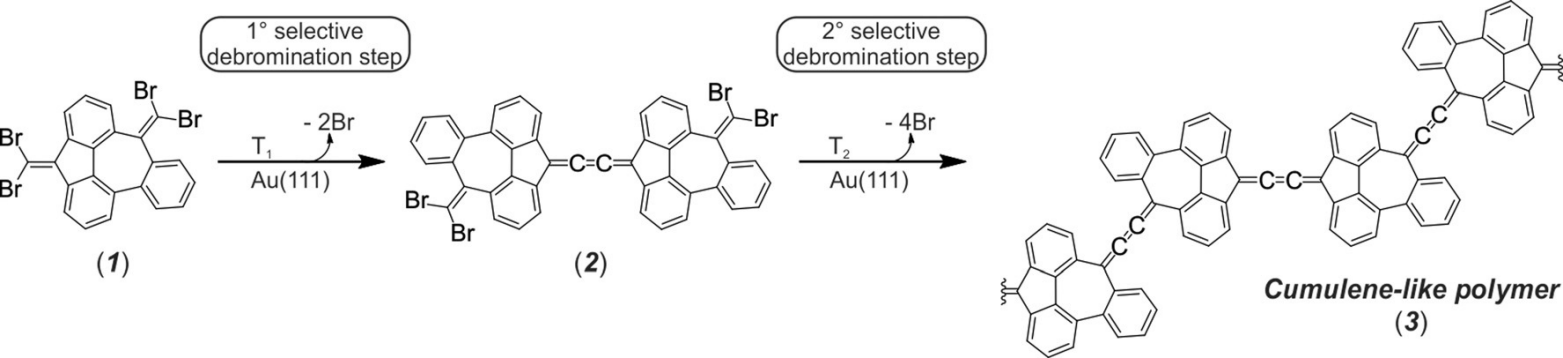
On‐surface synthesis of cumulene‐containing polymers.

The molecular precursor **1** consists of a tribenzoazulene (TBA) core functionalized with two dibromomethylenes at the pentagon and heptagon ends. It was synthesized in four steps: First, a Suzuki coupling of 2‐biphenylboronic acid and 2‐bromoisophthalaldehyde gave aldehyde **4** in a yield of 81 %, which was then quantitatively oxidized to the carboxylic acid **5**. In the next step, compound **5** was cyclized with methanesulfonic acid to ketone **6** with a yield of 72 % and finally precursor **1** was achieved by Ramirez reaction with a yield of 44 % (for synthetic details and characterization, see the Supporting Information). Scheme 1 illustrates the observed stepwise on‐surface reactions in the formation of polymers linked by cumulene‐like bonds (**3**) on a Au(111) substrate. A first annealing process leads to the partial dehalogenation of **1** which produces molecular dimers **2** that are selectively connected via three consecutive C−C double bonds linked to the nonbenzenoid pentagonal moieties. A second annealing process activates the next dehalogenation step, giving rise to 1D polymer chains **3** that link the azulene cores via the same cumulene‐like connections.

To investigate the on‐surface reactions depicted in Scheme 1, a sub‐monolayer coverage of **1** was sublimed onto the Au(111) substrate held at 325 K. Large‐scale STM images show sporadic individual molecular species with a large apparent height of 3.0 Å (measured at a sample bias of −0.9 V) coexisting with dimers of a similar apparent height for both or one of the molecular species (see Figure S1 for more details).

**Figure 1:**
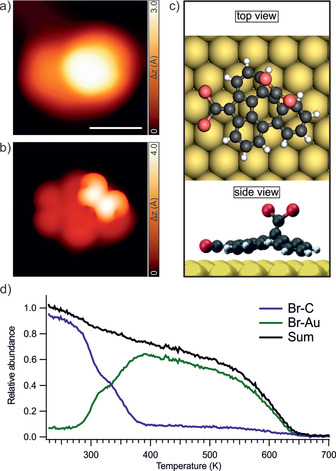
Identification of **1** after deposition on Au(111). a) High‐resolution STM image of an intact molecular precursor evidencing the highly nonplanar conformation of the molecule on the surface. *V*_b_=−1 V, *I*_t_=50 pA, scale bar=1 nm. b) DFT‐simulated STM image of the molecule highlighted in (a). c) Top (upper panel) and side (lower panel) views of the DFT equilibrium geometry of **1** on Au(111). d) Normalized intensity of the Br‐C (blue) and Br‐Au (green) components of the Br 3d signal, extracted from the corresponding TP‐XPS map, monitored in a temperature range of 230–700 K (see Figure S3 for further details).

These features suggest that the molecules adopt a highly nonplanar conformation on the surface. Figure 1 a shows a high‐resolution STM image of an intact molecule. The experimental features of **1** are well reproduced by the corresponding STM simulation based on the DFT‐optimized geometry on the Au(111) surface (Figure 1 b,c), which reveals that the two bromine atoms at the pentagon end of the TBA are much closer to the gold surface than those at the heptagon end (see Figure S2 for details regarding the possible adsorption geometries of **1**). Therefore, this adsorption geometry favors the sequential surface‐catalyzed dehalogenation of the molecular precursors, where the dibromomethylenes attached to the pentagonal moiety are debrominated first by virtue of their close proximity to the gold surface.

In order to unravel the reaction mechanism that leads to **3**, we have deposited **1** on Au(111) held at 20 K to prevent any activation of the molecular precursors, and performed temperature‐programmed XPS (TP‐XPS) measurements.^25^ We monitored the Br 3d core level during the annealing of the sample from 230 to 700 K with a constant heating rate of 0.2 K s^−1^ and extracted kinetic curves that clarify the chemical reactions involving bromine.^28^ Figure 1 d displays the relative amount of the two main components of the bromine signal, that is, Br‐C (due to intact bromine functionalities still anchored to the molecule) and Br‐Au (associated with bromine detached from the precursor and chemisorbed on the gold substrate). A two‐step sequential dehalogenation process is deduced from these curves, in agreement with the pathway described above (see Figure S3 for details about the TP‐XPS map that monitors the reaction pathway and high‐resolution XPS spectra). Due to the highly nonplanar conformation of **1** described above, we assume that in a first debromination step (from 270 to 320 K), the bromine atoms at the pentagon end of the TBA core dissociate from **1** and bind to the Au(111) surface, which induces the formation of **2**, while the second debromination step (from 320 to 390 K) is attributed to the dissociation of the bromine atoms on the heptagon end. At higher temperatures, the total bromine signal gradually decreases indicating bromine desorption from the Au(111) surface which is completed at 650 K.

**Figure 2:**
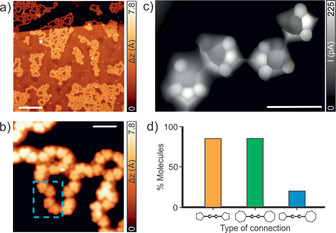
Formation of cumulene‐containing polymers on Au(111). a) Overview STM image showing the formation of meandering 1D chains after deposition of **1** and subsequent annealing at 400 K. b) High‐magnification STM image allowing the identification of triangular‐shaped molecular units within the polymers **3**. The blue rectangle highlights the nanostructure presented in (c). c) CO‐functionalized UHR‐STM image of **3** where the intramolecular features of the nonbenzenoid molecular backbone in the polymers are clearly discerned. d) Histogram depicting the selectivity observed in the C−C coupling between pentagonal–pentagonal, heptagonal–heptagonal, and pentagonal–heptagonal moieties. Scanning parameters: a) *V*_b_=−1 V, *I*_t_=100 pA. b) *V*_b_=0.1 V, *I*_t_=100 pA. c) Open feedback parameters: *V*_b_ *=*−5 mV, *I*_t_=50 pA. Scale bars: a) 20 nm, b) 2 nm, and c) 1 nm.

Debromination of **2** is completed after annealing the sample to 400 K, as can be seen in Figure 2 and Figure 1 d. At such temperatures, new species appearing as 1D chains with lower apparent height (1.6 Å) are discerned, which we assign to polymers linked by cumulene‐like bonds (**3**). Figure 2 a,b depicts constant‐current STM images of **3**, exhibiting meandering chain‐like structures composed of molecules with triangular appearance coexisting with a small fraction (<7 %) of side products (fused molecules), which are tentatively attributed to the formation of pentalene units (Figure S4).^27^ Figure 2 c shows an ultrahigh‐resolution STM (UHR‐STM) image of **3** acquired with a CO‐functionalized tip recorded in the Pauli repulsion regime,^28^, ^29^, ^30^ where the intramolecular features of the nonbenzenoid molecular backbone in the polymers are clearly discerned. Importantly, a selective C−C coupling, that is, pentagon–pentagon and heptagon–heptagon connections, in the formation of **3** is observed. The graph depicted in Figure 2 d displays the selectivity in the molecular connections of the polymers. Specifically, more than 80 % of the connections occur between pentagonal–pentagonal or heptagonal–heptagonal moieties (Scheme 1), while all other connections take place between pentagonal–heptagonal moieties (statistics out of >110 molecules). Such selectivity induces a hierarchical C−C coupling which is often hampered in covalent linking reactions obtained by one‐step on‐surface reactions, where only rather simple nanostructures have been achieved.

**Figure 3:**
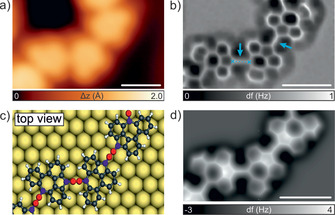
nc‐AFM study of **3**. a) High‐resolution STM image showing a segment of a polymer **3**. *V*_b_=−1 V, *I*_t_=100 pA. Scale bar: 1 nm. b) Constant‐height frequency‐shift nc‐AFM image of (a) acquired with a CO‐functionalized tip (*z* offset −35 pm below STM set point: 5 mV, 100 pA). The blue arrows highlight the cumulene bonds in **3**. Scale bar: 1 nm. c) Top view of the DFT equilibrium geometry of the polymer on Au(111) shown in (b). Colored red and blue carbon atoms indicate the central and adjacent double bonds, respectively, in **3**. d) Simulated nc‐AFM image of (b). Scale bar: 1 nm.

The use of similar functional groups in the on‐surface formation of cumulene‐like bridged dimers^20^ and polymers,^21^ and ethynylene‐bridged polymers^31^ on the Au(111) surface has recently been investigated. In order to confirm the chemical nature of the connections observed in **3**, nc‐AFM measurements using a CO‐functionalized tip were performed.^33^ Figure 3 a,b depicts the resulting STM and constant‐height frequency‐shift images where features assigned to the nonbenzenoid molecular backbone linked by a sharp line with a homogeneous contrast are clearly resolved. Importantly, intramolecular contrast observed in nc‐AFM images results from the short‐ranged Pauli repulsion being maximized in the areas of highest electron density. Therefore, minor variations in electron density assigned to specific bond orders can be recognized.^32^, ^33^ For instance, C−C triple bonds exhibit in nc‐AFM images an enhanced contrast at their central positions.^31^, ^34^, ^35^, ^36^, ^37^ Notably, the sharp lines observed in the intermolecular connections of **3** (highlighted by the blue arrows in Figure 3 b) are well reproduced by the simulated nc‐AFM image depicted in Figure 3 d, (see Figure S5 for nc‐AFM images acquired at different tip–polymer distances). In addition, the DFT‐optimized structure (Figure 3 c) of **3** on Au(111) reveals an adsorption height of 3.3 Å, with a C−C distance between cumulene bonds of 3.9 Å (in agreement with the experimentally measured length of 4.0±0.2 Å, dashed blue line in Figure 3 b). Altogether, this provides conclusive evidence for the structural assignment of the three consecutive C−C double bonds,^20^, ^22^ that is, cumulene‐like connections in the formation of **3** on the Au(111) surface. In order to further investigate the bond orders of the cumulene‐like connections, we performed bond order analysis on the DFT electronic structure based on Bader's charge density partitioning.^39^, ^40^ We find that the bond order of the middle bond is 2.11 (C−C distance of 1.32 Å, colored red in Figure 3 c), while the bond order of the neighboring bonds is 1.44 (C−C distance of 1.41 Å, colored blue in Figure 3 c), indicating a stronger similarity to a cumulene system, rather than a configuration involving a triple bond.^40^

**Figure 4:**
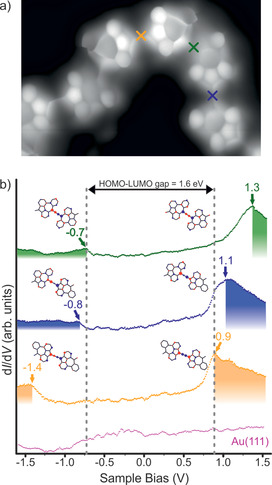
Electronic properties of **3** on Au(111). a) UHR‐STM image of **3**. The orange, green, and blue crosses indicate the positions where differential conductance d*I*/d*V* spectra were acquired. Open feedback parameters: *V*_b_=−5 mV, *I*_t_ *=*50 pA. b) d*I*/d*V* spectra acquired at the pentagon–cumulene–pentagon (orange line), pentagon–cumulene–heptagon (blue line) and heptagon–cumulene–heptagon (green line) connections of **3**, revealing a HOMO–LUMO gap of 1.6 eV with the HOMO (LUMO) localized on the heptagon–cumulene–heptagon (pentagon–cumulene–pentagon) connections. The reference spectrum taken on the bare Au(111) surface is shown in pink. The insets show the frontier orbital wave functions of the corresponding molecular dimers (see Figure S6 for details).

STS measurements on **3** were performed in order to probe its electronic structure. The positive‐ and negative‐ion resonances (PIR and NIR) that derive from the HOMO and LUMO of the polymer are experimentally detected at −0.7 eV and 0.9 eV, respectively (Figure 4). Therefore, the HOMO–LUMO gap of the polymer on Au(111) is found to be 1.6 eV. Interestingly, the voltage‐dependent differential conductance spectra (d*I*/d*V* vs. *V*) acquired at the three different cumulene connections (i.e. pentagon–pentagon, pentagon–heptagon, and heptagon–heptagon) reveal a systematic upward shift of the frontier orbital energy positions from pentagon–pentagon to heptagon–heptagon connections. This originates from the frontier orbital energy ordering of the singular unit of the polymer, whose HOMO−1 and LUMO are localized at the pentagon edge, while HOMO and LUMO+1 are localized at the heptagon, as shown by tight binding (TB) calculations (see Figure S6 for detailed TB calculations and additional d*I*/d*V* spectra). The pairwise coupling of these orbitals form the experimentally observed frontier states of the polymer which are strongly localized on the cumulene connections. Detailed analysis reveals that the frontier orbitals at the pentagon–pentagon connection are the antibonding coupling of two HOMO−1 and the bonding coupling of two LUMO orbitals of the singular unit, which are both lower in energy than the corresponding orbitals in the pentagon–heptagon connection (antibonding HOMO−1 and HOMO; and bonding LUMO and LUMO+1). The corresponding frontier orbitals for the heptagon–heptagon connection (antibonding of two HOMO and bonding of two LUMO+1) are in turn highest in energy. As a result of these couplings, the HOMO and LUMO of **3** are localized at the pentagon–pentagon and heptagon–heptagon connections, respectively. This is further corroborated by DFT band structure calculation for the polymer with alternating pentagon–pentagon and heptagon–heptagon connections, which shows flat bands (Figure S7), revealing the nondispersive nature of the polymers, as expected from the obtained localized states.

## Conclusion

We have demonstrated the on‐surface synthesis of a polymer linked via cumulene‐like bonds on a coinage metal surface by a combination of high‐resolution STM and nc‐AFM imaging together with DFT calculations, STS, and XPS. A sequential thermally induced dehalogenation is explained by the preferred geometry that the molecular precursor adopts upon absorption on the gold surface, which gives rise to selective C−C coupling, that is, pentagon–pentagon and heptagon–heptagon connections, in the formation of **3**. The formation of linear cumulene‐like connections between the nonbenzenoid tribenzoazulene backbone units of the polymer **3** is confirmed by Bader analysis and nc‐AFM investigations. In addition, STS studies together with theoretical calculations reveal that **3** exhibits an electronic gap of 1.6 eV where a systematic shift of the frontier orbitals from pentagon–cumulene–pentagon to heptagon–cumulene–heptagon is observed. We expect that our study is of general relevance for the synthesis and characterization of carbon allotropes with sp‐hybridization, as well as for the stepwise formation of carbon–carbon covalent bonds on surfaces, thus opening new avenues in the field of on‐surface synthesis with prospects for applications in molecular electronics.

## Experimental Section

Sample preparation and STM/nc‐AFM measurements. Experiments were performed under ultrahigh‐vacuum conditions (base pressure below 5×10^−10^ mbar) with a low‐temperature STM/AFM Scienta Omicron scanning probe microscope. The Au(111) substrate was prepared by repeated cycles of Ar^+^ sputtering (*E*=1 keV) and subsequent annealing to 750 K for 15 minutes. All STM images shown were recorded in constant‐current mode with electrochemically etched tungsten tips at a sample temperature of 5 K. Scanning parameters are specified in each figure caption. The molecular precursor **1** was thermally deposited onto the clean Au(111) surface held at room temperature with a typical deposition rate of 0.4 Å min^−1^ (sublimation temperature ≈420 K). Nc‐AFM measurements were performed with a tungsten tip attached to a tuning fork sensor.^41^ The tip was a posteriori functionalized by the controlled pick‐up of a single CO molecule at the tip apex from the previously CO‐dosed surface. The functionalized tip enables the imaging of the intramolecular structure of organic molecules.^42^ The sensor was driven at its resonance frequency (22 350 Hz) with a constant amplitude of ≈70 pm. The shift in the resonance frequency of the tuning fork (with the attached CO‐functionalized tip) was recorded in constant‐height mode (Omicron Matrix electronics and HF2Li PLL by Zurich Instruments). The STM and nc‐AFM images were analyzed using WSxM.^43^

XPS measurements were performed at the X03DA beamline (PEARL endstation) at the SLS synchrotron radiation facility (Villigen, Switzerland), using linearly (and partially circularly left/right) polarized radiation with photon energy of 425 eV. XPS spectra were obtained in normal emission geometry, using a hemispherical electron analyzer equipped with a multichannel plate (MCP) detector. HR‐XPS spectra were recorded at the indicated temperature in “swept” mode with 20 eV pass energy, while the TP‐XPS measurement was performed during the heating of the sample (constant heating rate of 0.2 K s^−1^) using the “fixed” mode (snapshots of the Br 3d core level) acquiring each spectrum for 5 s with 100 eV pass energy. The TP‐XPS maps have a resolution of 3.5 °C in temperature and 17 s in time.

DFT calculations were performed with the CP2K code (freely available at http://www.cp2k.org/)^44^ utilizing the AiiDA platform.^46^ The electronic states were expanded with a TZV2P Gaussian basis set^47^ for C and H species and a DZVP basis set for Au species. A cutoff of 600 Ry was used for the plane wave basis set. We used Norm Conserving Goedecker–Teter–Hutter^48^ pseudopotentials and the PBE^49^ exchange‐correlation functional with the D3 dispersion corrections proposed by Grimme.^50^ The surface/adsorbate systems were modelled within the repeated slab Scheme, that is, a simulation cell containing four atomic layers of Au along the [111] direction and a layer of hydrogen atoms to passivate one side of the slab in order to suppress one of the two Au(111) surface states. 40 Ångstroms of vacuum were included in the simulation cell to decouple the system from its periodic replicas in the direction perpendicular to the surface. We considered supercells of 41.27×40.85 Å corresponding to 224 surface units. To obtain the equilibrium geometries, we kept the atomic positions of the bottom two layers of the slab fixed to the ideal bulk positions; all other atoms were relaxed until forces were lower than 0.005 eV Å^−1^. To obtain simulated STM images within the Tersoff–Hamann approximation,^51^ we extrapolated the electronic orbitals obtained from CP2K to the vacuum region in order to correct the wrong decay in vacuum of the charge density due to the localized basis.

The Probe Particle model^50^ was used to simulate AFM images. A two‐point implementation of the model, where two probe particles represent the carbon and oxygen atoms in the CO molecule, has been employed. The stiffness parameters of the Probe Particle as well as the Lennard–Jones parameters of the tip were obtained by fitting to DFT calculations for the polymer **3** depicted in Figure 3 c. The charges of the tip atoms were assigned by the restrained electrostatic potential method.^51^ For the tip sample electrostatic interactions the Hartree potential obtained from the CP2K slab calculations was used.

Bond order analysis was performed by the scheme introduced by Ángyán et al.^38^ based on the Kohn–Sham orbitals obtained from the CP2K calculation of the whole polymer‐slab system. Bader's basins were calculated with the Bader Charge Analysis code by Henkelman et al.^52^ based on the CP2K valence charge augmented with frozen core charge density.

Band structures were calculated with the Quantum Espresso software package using the PBE exchange‐correlation functional. The plane wave basis with an energy cutoff of 400 Ry for the charge density was used together with PAW pseudopotentials (SSSP)^53^ and a Monkhorst k‐mesh of 13×1×1.

## Conflict of interest

The authors declare no conflict of interest.

## Supporting information

As a service to our authors and readers, this journal provides supporting information supplied by the authors. Such materials are peer reviewed and may be re‐organized for online delivery, but are not copy‐edited or typeset. Technical support issues arising from supporting information (other than missing files) should be addressed to the authors.

Supplementary
