# Supplementary material for: On‐Surface Synthesis of Cumulene‐Containing Polymers via Two‐Step Dehalogenative Homocoupling of Dibromomethylene‐Functionalized Tribenzoazulene
Source: Angew Chem Int Ed Engl. 2020 May 29;59(32):13281–7. doi: 10.1002/anie.202001939 (PMC7496152; doi:10.1002/anie.202001939)
Supplement: Supplementary file 1 — Supplementary [file ANIE-59-13281-s001.pdf]

Angewandte Chemie
Eine Zeitschrift der Gesellschaft Deutscher Chemiker

Supporting Information

On-Surface Synthesis of Cumulene-Containing Polymers via Two-Step Dehalogenative Homocoupling of Dibromomethylene-Functionalized Tribenzoazulene

José I. Urgel, Marco Di Giovannantonio, Kristjan Eimre, Thorsten G. Lohr, Junzhi Liu, Shantanu Mishra, Qiang Sun, Amogh Kinikar, Roland Widmer, Samuel Stolz, Max Bommert, Reinhard Berger, Pascal Ruffieux, Carlo A. Pignedoli, Klaus Müllen, Xinliang Feng,* and Roman Fasel**

anie_202001939_sm_miscellaneous_information.pdf

Supporting Information

©Wiley-VCH 2016

69451 Weinheim, Germany

Abstract: Cumulene compounds are notoriously difficult to prepare and study due to dramatically increasing reactivity with increasing number of consecutive double bonds. In this respect, the emerging field of on-surface synthesis provides exceptional opportunities because it relies on reactions on clean metal substrates under well-controlled ultrahigh vacuum conditions. Here we report the on-surface synthesis of a polymer linked by cumulene-like bonds on a Au(111) surface via sequential thermally activated dehalogenative C-C coupling of a tribenzo-azulene precursor equipped with two dibromomethylenes. The structure and electronic properties of the resulting polymer with cumulene-like pentagon-pentagon and heptagon-heptagon connections have been investigated by means of scanning probe microscopy and spectroscopy methods and X-ray photoelectron spectroscopy, complemented by density functional theory calculations. Our results provide perspectives for the on-surface synthesis of compounds containing cumulene-like bonds, as well as protocols relevant to the stepwise fabrication of carbon-carbon bonds on surfaces.

DOI: 10.1002/anie.202001939

Table of Contents

1. **Synthesis of precursor 1**
2. **Additional experimental results**
3. **Mass spectra**
4. **NMR spectra**

1. Synthesis of precursor 1

General information

Unless otherwise stated, commercially available starting materials, catalysts, reagents, and dry solvents were used without further purification. Reactions were performed using standard vacuum-line and Schlenk techniques. All the starting materials were obtained from Sigma Aldrich, TCI, abcr, Alfa Aesar, Acros Organics, Sigma Aldrich or chemPUR. Catalysts were purchased from Strem.

Column chromatography was performed on silica (SiO_2, particle size 0.063−0.200 mm, purchased from VWR). Silica-coated aluminum sheets with a fluorescence indicator (TLC silica gel 60 F_{254}, purchased from Merck KGaA) were used for thin layer chromatography.

High-resolution ESI and APCI mass spectra were recorded with an Agilent 6538 UHD accurate-mass Q-TOF LC-MS system using the positive and negative mode.

NMR data were recorded on a Bruker AV-II 300 spectrometer operating at 300 MHz for ^{1}H and 75 MHz for ^{13}C, on a Bruker™ AV-III 600 spectrometer operating at 600 MHz for ^{1}H and 151 MHz for ^{13}C and on a Bruker Ascend 300 spectrometer operating at 300 MHz for ^{1}H and 76 MHz for ^{13}C with standard Bruker pulse programs at room temperature (296 K). Chemical shifts were referenced to δ_{TMS} = 0.00 ppm (^{1}H, ^{13}C). Chemical shifts (δ) are reported in ppm. Coupling constants (J) are reported in Hz. Dichloromethane-d_2 ($\delta(^1H)$ = 5.32 ppm, $\delta(^{13}C)$ = 53.8 ppm), tetrachloroethane-d_2 ($\delta(^1H)$ = 5.91 ppm, $\delta(^{13}C)$ = 74.2 ppm), chloroform-d_1 ($\delta(^1H)$ = 7.26 ppm, $\delta(^{13}C)$ = 77.2 ppm) or methanol-d_4 ($\delta(^1H)$ = 3.31 ppm, $\delta(^{13}C)$ = 49.0 ppm) were used as solvents. The following abbreviations are used to describe peak patterns as appropriate: s = singlet, d = doublet, t = triplet, q = quartet, and m = multiplet. Dichloromethane-d_2 (99.9 atom% D) was purchased from Euriso-top, chloroform-d_1 (99.8 atom% D) and methanol-d_4 were purchased from Deutero GmbH.

Melting points were determined on a Büchi Melting Point M-560 in a range of 50−400 °C with a temperature rate of 10 °Cmin^{-1}.

[1,1':2',1''-terphenyl]-2,6-dicarbaldehyde (**4**)

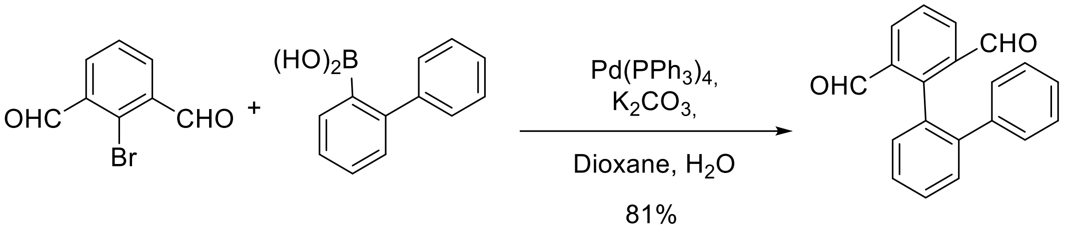

A 500 mL round-bottom Schlenk flask was charged with 2-bromoisophthalaldehyde (6.0 g, 26.76 mmol), 2-biphenylboronic acid (12.8 g, 61.54 mmol), K_2CO_3 (25.5 g, 184.62 mmol) and $Pd(PPh_3)_4$ (0.6 g, 0.48 mmol) and was evacuated and charged with argon three times. Then degassed 1,4-dioxane (300 mL) and water (30 mL) were added and the reaction was heated at 95 °C for 3 days under argon atmosphere. After cooling to room temperature, the mixture was poured into water and extracted with DCM three times. The organic layer was washed with brine and dried over anhydrous $MgSO_4$. The solvent was removed under vacuum. The residue was purified by silica gel column chromatography (ethyl acetate:cyclohexane = 1:20 to 1:10) and washed with ethanol to give compound as a white solid (6.2 g, yield: 81 %).

mp: 160°C; R_f: 0.41 (ethyl acetate:isohexane = 1:5); ^{1}H NMR (600 MHz, CD_2Cl_2) δ (ppm) = 9.81 (d, *J* = 0.6 Hz, 2H), 8.07 (d, *J* = 7.7 Hz, 2H), 7.62 (td, *J* = 7.6, 1.3 Hz, 1H), 7.57 – 7.49 (m, 3H), 7.41 (dd, *J* = 7.6, 1.0 Hz, 1H), 7.18 – 7.13 (m, 3H), 7.02 – 6.98 (m, 2H); ^{13}C NMR (151 MHz, CD_2Cl_2) δ (ppm) = 190.95, 147.80, 143.10, 140.26, 135.11, 132.79, 132.30, 131.58, 130.64, 129.98, 129.81, 128.68, 128.65, 127.78, 127.57; HR-MS (APCI, pos.) *m/z*: $[(M+H)^+]$ calcd for $C_{20}H_{14}O_2$: 287.1067; found: 287.1066; error -0.35 ppm; elemental analysis calcd for $C_{20}H_{14}O_2$: C 83.90; H 4.93; found C 83.90; H 4.89.

[1,1':2',1''-terphenyl]-2,6-dicarboxylic acid (**5**)

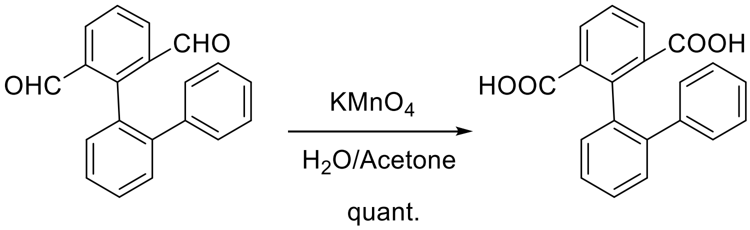

Compound **4** (2.0 g, 7 mmol) was solved in acetone (75 mL) and a suspension of $KMnO_4$ (2.4 g, 15.4 mmol) in water (95 mL) was added in one portion and the mixture was stirred overnight. The solvent was removed, dissolved in a 1:1 mixture of DCM:MeOH and filtered through a plug of celite. After the solvent was removed, the carboxylic acid **5** was yield as white solid (2.2 g, quant.).

mp: 288-290°C; R_f: 0.43 (methanol:DCM = 1:10); ^{1}H NMR (300 MHz, CD_3OD) δ (ppm) = 7.49 (d, *J* = 7.6 Hz, 2H), 7.40 – 7.33 (m, 3H), 7.29 – 7.15 (m, 4H), 7.04 (m, 3H); ^{13}C NMR (75 MHz, CD_3OD) δ (ppm) = 176.20, 143.13, 142.68, 140.89, 140.66, 139.26, 131.51, 131.16, 130.33, 129.68, 128.30, 127.76, 127.21,

127.12; HR-MS (APCI, pos.) *m/z*: [(M+H)$^+$] calcd for $C_{20}H_{14}O_4$: 319.0954; found: 319.0965; error: +3.45 ppm.

benzo[5,6]cyclohepta[1,2,3,4-def]fluorene-1,5-dione (**6**)

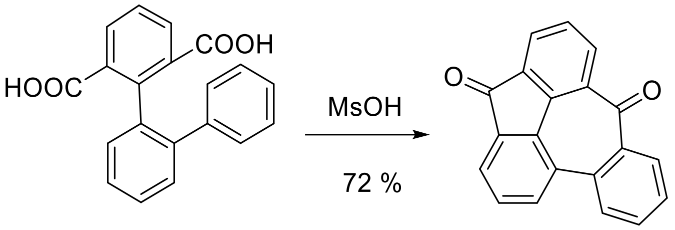

A 1 L round flask with condenser was charged with carboxylic acid **5** (9.2 g, 28.8 mmol) and methanesulfonic acid (300 mL) and heated to 80 °C for 6 h. After cooling to room temperature, the mixture was slowly poured on ice water and the resulting precipitation was filtered and washed with water, saturated $NaHCO_3$ solution and MeOH. The crude product was purified by silica gel column chromatography (ethyl acetate:cyclohexane = 1:5 to 1:1) to give compound **6** as a yellow solid (5.8 g, yield: 72 %).

mp: 225°C; R_f: 0.30 (ethyl acetate:cyclohexane = 1:5); ^{1}H NMR (300 MHz, CD_2Cl_2) δ (ppm) = 8.24 (dd, *J* = 7.9, 1.6 Hz, 1H), 8.22 – 8.15 (m, 2H), 8.10 (d, *J* = 8.1 Hz, 1H), 7.94 (dd, *J* = 7.2, 1.1 Hz, 1H), 7.83 – 7.73 (m, 2H), 7.68 – 7.62 (m, 1H), 7.62 – 7.52 (m, 2H); ^{13}C NMR (151 MHz, CD_2Cl_2) δ (ppm) = 192.31, 191.65, 143.18, 141.14, 139.74, 135.46, 135.29, 135.00, 134.93, 134.25, 134.16, 132.92, 132.48, 130.96, 130.49, 129.60, 129.27, 128.47, 124.50; HR-MS (APCI, pos.) *m/z*: [(M+H)$^+$] calcd for $C_{20}H_{11}O_2$: 283.0754; found: 283.0753; error: -0.18.

1,5-bis(dibromomethylene)-1,5-dihydrobenzo[5,6]cyclohepta[1,2,3,4-*def*]fluorene (**1**)

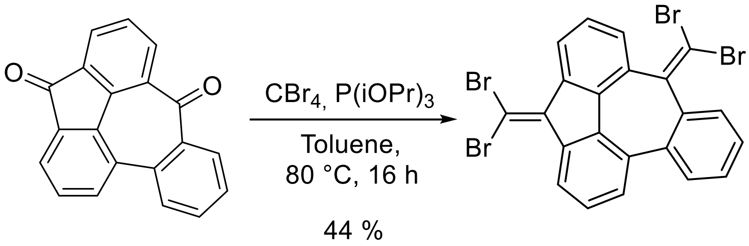

Compound **6** (800 mg, 2.83 mmol) and CBr_4 (10.3 g, 31.17 mmol) was added to a 500 mL Schlenk flask and the flask was evacuated and charged with argon three times. Then dry, degassed toluene (120 mL) was added and the mixture was cooled to 0 °C. At this temperature P(*i*OPr)$_3$ (10.30 mL, 39.67 mmol) was added and the mixture was stirred for 10 min. Afterwards, the cooling bath was removed and the mixture was heated to 80 °C for 15 h. The mixture was cooled to room temperature and the solvent was removed. The crude product was purified by silica gel column chromatography (DCM:cyclohexane = 1:8 to 1:6) to give compound **1** as a yellow solid (742 mg, yield: 44 %).

mp: 196°C (dec.); R_f: 0.65 (ethyl acetate:isohexane = 1:10); ^{1}H NMR (600 MHz, $CDCl_3$) δ (ppm) = 8.70 (dd, *J* = 7.9, 0.5 Hz, 1H), 8.64 (dd, *J* = 8.0, 0.7 Hz, 1H), 7.92 (d, *J* = 7.8 Hz, 1H), 7.66 – 7.62 (m, 2H), 7.58 – 7.55 (m, 1H), 7.50 (t, *J* = 7.9 Hz, 1H), 7.47 – 7.41 (m, 3H); ^{13}C NMR (151 MHz, $CDCl_3$) δ (ppm) = 143.68, 138.94, 138.83, 137.80, 137.78, 137.21, 134.89, 134.31, 133.70, 130.21, 129.90, 128.87, 128.72, 128.32, 128.16, 127.68, 127.53, 125.26, 124.95, 94.35, 91.92; HR-MS (APCI, pos.) *m/z*: $[(M+H)^+]$ calcd for $C_{22}H_{11}Br_4$: 594.7549; found: 594.7567; error: -3.03 ppm; $[(M)^+]$ calcd for $C_{22}H_{10}Br_4$: 593.7469; found: 593.7433; error: -6.06 ppm.

2. Additional experimental results

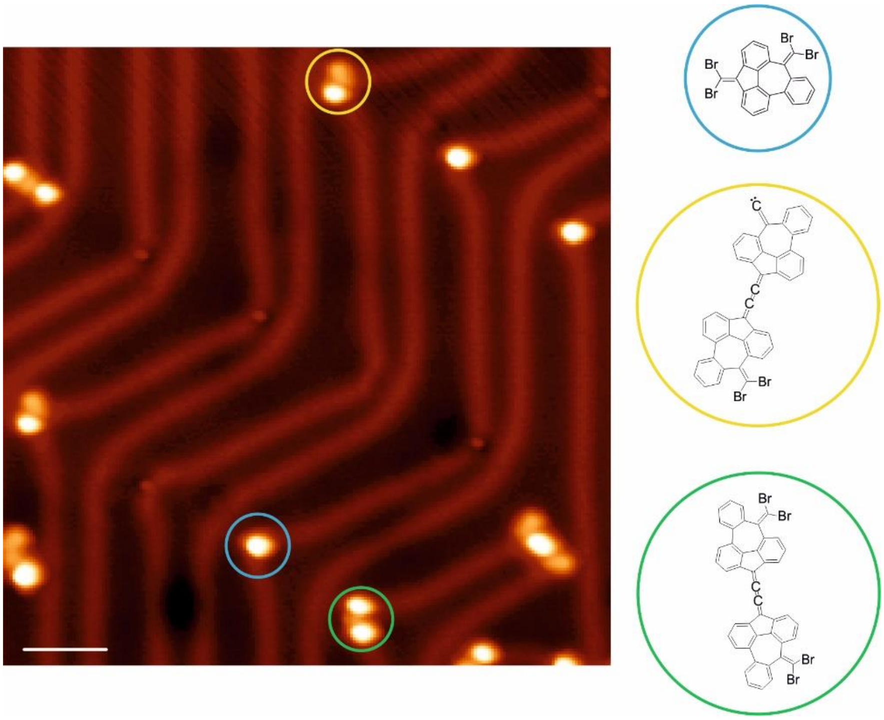

Figure S1. Deposition of low coverages of (**1**) onto the Au(111) substrate held at 300–325 K. The STM image shows intact as well as partially reacted molecular precursors. The blue circle highlights an intact molecular precursor, the green circle a dimer where the cleavage of four bromine atoms (two per molecule) and subsequent bond formation has taken place at one end, and the yellow circle shows a dimer where only one edge of two connected molecules remains with the two bromine atoms. V_b = -0.9 V, I_t = 30 pA. Scale bar: 5 nm.

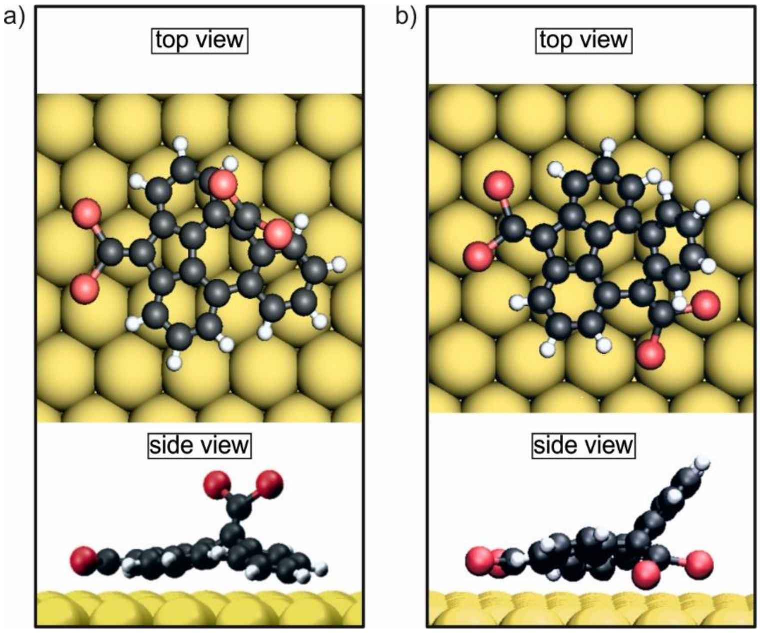

Figure S2. Top and side views of two different DFT equilibrium geometries of the molecular precursor (**1**) on the Au(111) surface. a) For this adsorption geometry, the two bromine atoms on the pentagon end of the TBA are much closer to the gold surface (3.3 Å) than those on the heptagon end of the TBA (6.5 Å) which are pointing away from the Au surface. b) In this case, the two bromine atoms on the pentagon end of the TBA are again at 3.3 Å, while the ones close to the heptagon end of the TBA are at 2.7 Å with respect to the gold surface. The geometry shown in (a) is preferred over the one depicted in (b) by 0.22 eV..

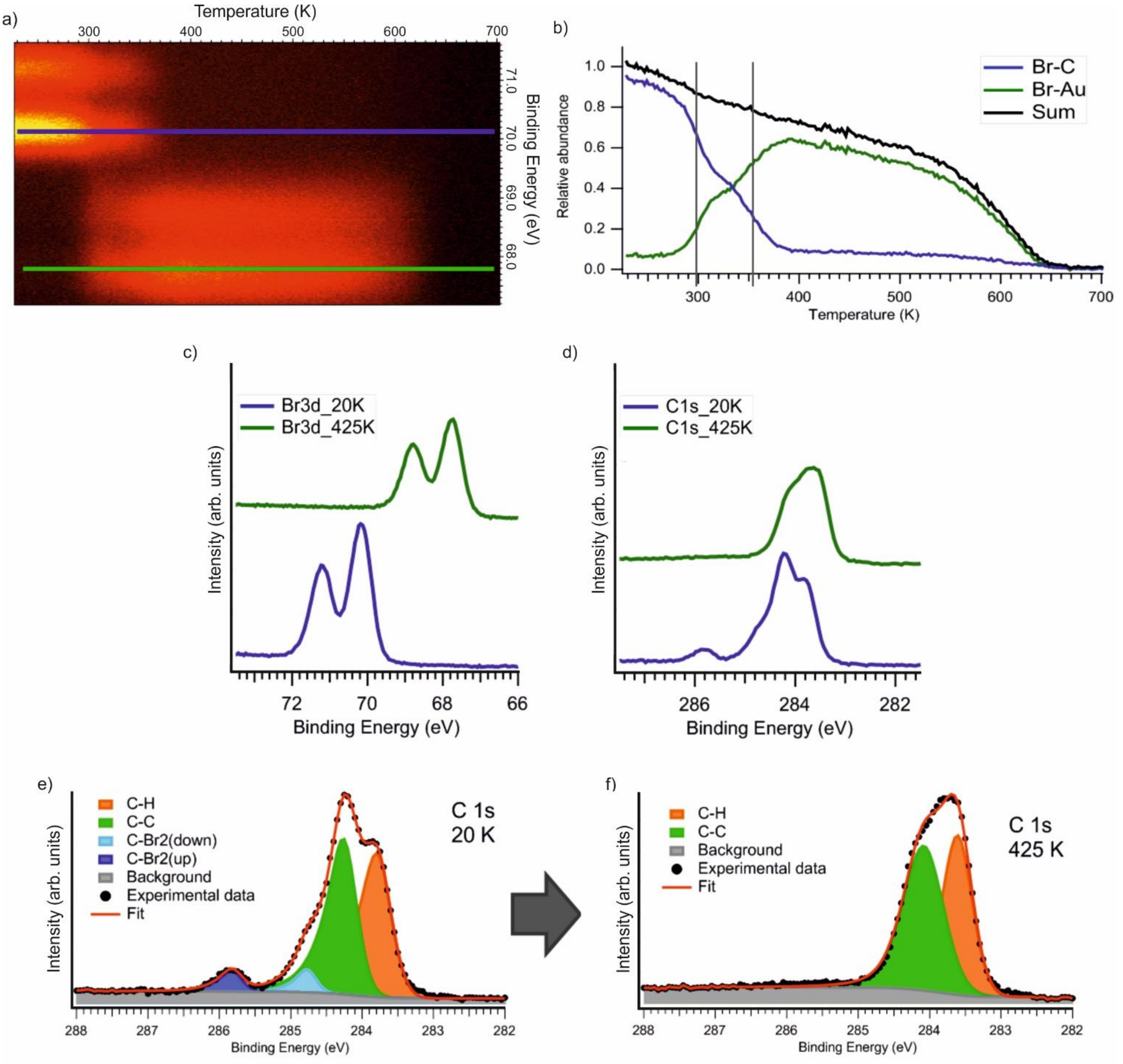

g)

Name	BE position	FWHM	Area	% Conc.
C-H	283.80	0.473	13775.0	45.45
C-C	284.26	0.436	13775.0	45.45
C-Br(up)	285.83	0.331	1377.5	4.55
C-Br(down)	284.78	0.301	1377.5	4.55

Name	BE position	FWHM	Area	% Conc.
C-H	283.61	0.485	13758.1	45.45
C-C	284.08	0.642	16509.7	54.55

Figure S3. Temperature programmed XPS (TP-XPS) measurements and HR-XPS investigations of the chemical transformations from 1 to 3. a) TP-XPS measurements map acquired at the Br 3d core level during the annealing (heating rate of 0.2 K·s^{-1}) of the Au(111) surface after deposition of **1** at 20 K. The Br 3d doublet undergoes a chemical shift at about 300-350 K. b) Line profiles extracted from the map in a) at binding energy values of 70.1 eV (Br 3d$_{5/2}$ of Br-C doublet) and 67.8 eV (Br 3d$_{5/2}$ of Br-Au doublet). These curves represent the kinetics of the debromination process (i.e. Br detaches from **1** and chemisorbs onto the gold substrate) and show that debromination takes place in two distinguishable steps, centered at about 300 K and 350 K, respectively. The debromination at lower temperature is associated to the removal of bromine atoms from the pentagonal moiety of **1** while the process occurring at higher temperature is due to the removal of bromine atoms from the heptagonal moiety of **1**. The blue and green curves represent the Br-C and Br-Au components, respectively, while the black curve is their sum. The desorption of bromine atoms from Au(111) is completed at about 650 K. c,d) High-resolution XPS spectra acquired at the Br 3d and C 1s core levels, respectively, on the Au(111) surface after deposition of **1** at 20 K and after annealing to 425 K. The C 1s signals have been deconvoluted and the corresponding fits are reported in panels e,f), where a Shirley background was used. Interestingly, the two different C-Br functional groups at the pentagonal (down) and heptagonal (up) moieties of **1** produce distinct spectroscopic signals, at 284.8 eV and 285.8 eV, respectively. g) Parameters resulting from the fit of the two C 1s spectra.

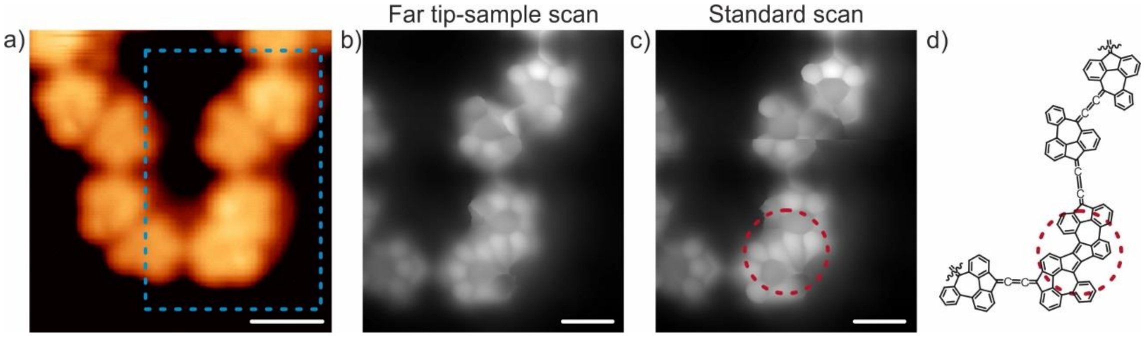

Figure S4. Side reactions observed for (**3**). a) Zoom-in STM image allowing the identification of triangular-shaped molecular units within the polymers coexisting with fused molecules which are tentatively attributed to the formation of pentalene units. The blue rectangle highlights the region presented in (b,c). b,c) CO-functionalized UHR-STM images of (**3**) where the intramolecular features of the nonbenzenoid molecular backbone are clearly discerned. The dashed red circle in (c) highlights the formation of a pentalene unit. d) Structural model of (c). Scanning parameters: a) V_b = 0.1 V, It = 100 pA. b, c) Open feedback parameters: V_b = −5 mV, I_t = 100 pA. Scale bars: (a) 1 nm, (b,c) 0.5 nm.

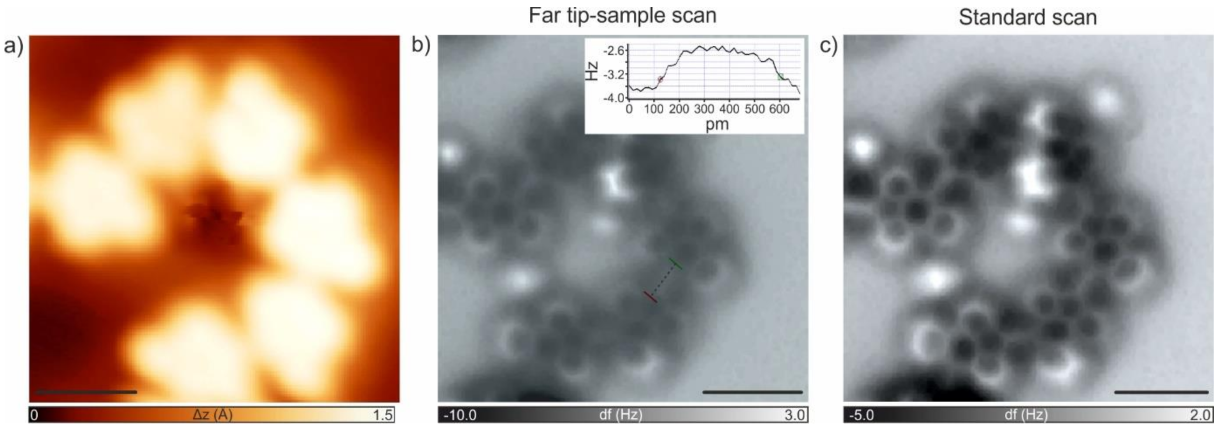

Figure S5. Constant-height frequency-shift nc-AFM study at two tip-sample distances. a) High-resolution STM image showing a segment of a polymer (3). V_b = 20 mV, I_t = 100 pA. Scale bar: 1 nm. b,c) Constant-height frequency-shift nc-AFM image of panel (a) acquired with a CO-functionalized tip. The tip-sample distances in (b) and (c) differ by 20 pm. In (b), the typical bright protrusion that appears for a triple bond connection is not observed (see the 1D frequency shift profile inset of the link between molecular units), which further confirms that the connections between the non-benzenoid polymer units are cumulene-like.

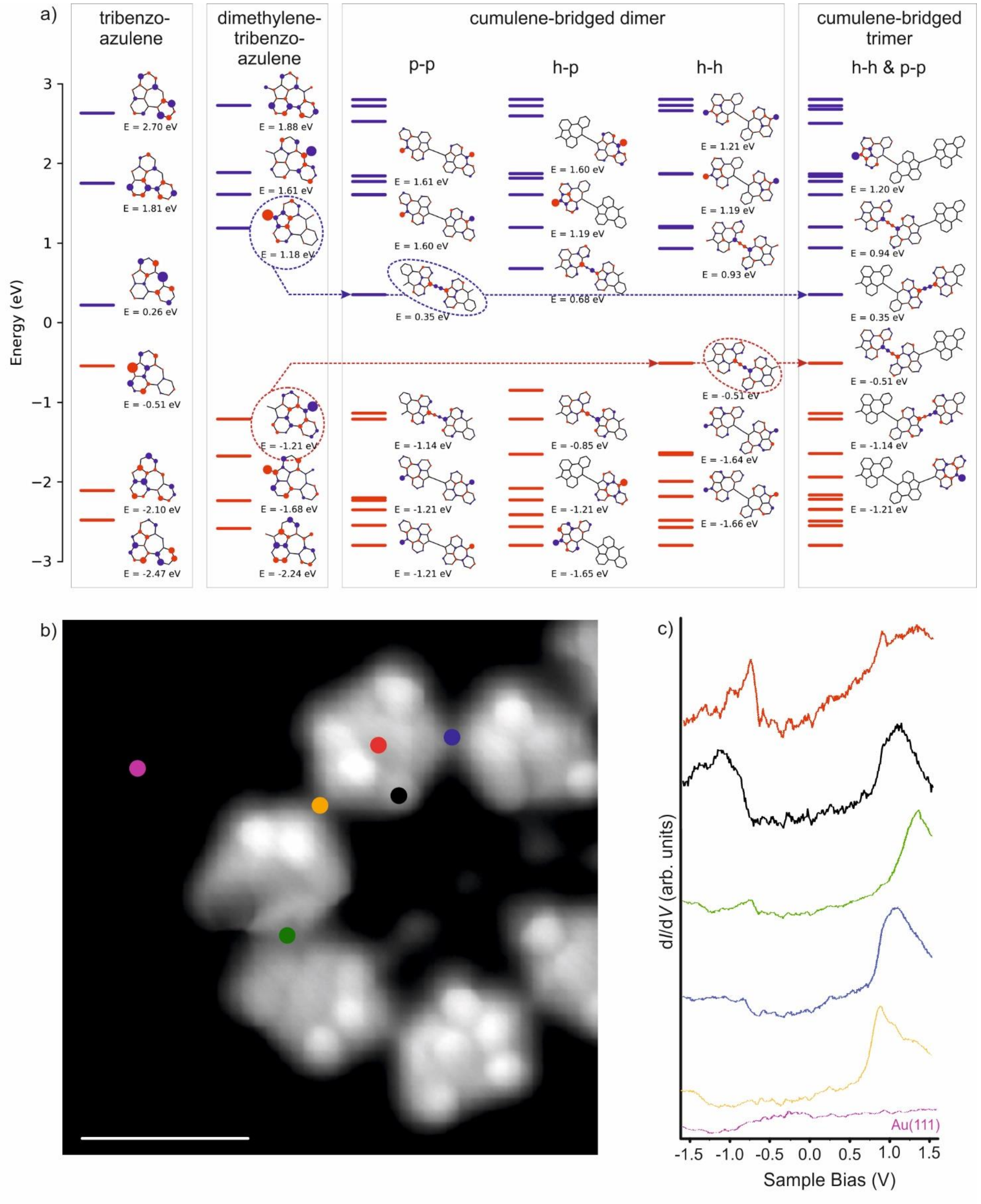

d)

d*I*/d*V* spectra	HOMO (eV)	LUMO (eV)	d*I*/d*V* spectra	HOMO (eV)	LUMO (eV)
pentagon-pentagon	-1.4	0.9	center of the molecule	-0.8	0.9
pentagon-heptagon	-0.8	1.0	edge of the molecule	-1.1	1.1
heptagon-heptagon	-0.7	1.3			

Figure S6. Electronic properties of **3**. a) Frontier orbital energies and wave functions for finite sub-systems of the polymer (from left: singular unit without the connecting carbons (tribenzo-azulene); singular unit (dimethylene-tribenzo-azulene); dimers with pentagon-pentagon (p-p), heptagon-pentagon (h-p) and heptagon-heptagon (h-h) connections; trimer with heptagon-heptagon & pentagon-pentagon (h-h & p-p) connections) calculated with the nearest neighbor tight binding (TB) model with the hopping parameter of -2.7 eV. Compared to the tribenzo-azulene, the dimethylene-tribenzo-azulene has four frontier orbitals localized at the connection sites: HOMO-1 and LUMO at the pentagon edge; and HOMO and LUMO+1 at the heptagon edge. Coupling two of these units together will result in a dimer system with the HOMO being the anti-bonding orbital of the coupled occupied edge-states and LUMO the bonding orbital of the coupled unoccupied edge-states. As the singular unit edge-state amplitude at the connection sites is similar, so is the hybridization energy of the three possible connections, and therefore the resulting orbital energy ordering is determined by the hybridized orbital energy locations. This produces the trend that the frontier orbitals corresponding to the pentagon-pentagon connection are shifted lowest and the heptagon-heptagon are shifted highest in energy. The trimer shows that the localized frontier states are not affected by further lengthening of the polymer and the HOMO and LUMO are inherited from the corresponding dimers. b) UHR-STM image of **3** acquired with a CO functionalized tip, where the intramolecular features of the nonbenzenoid molecular backbone are discerned. Open feedback parameters: $V_b = -5$ *mV*, $I_t = 50$ *pA*. Scale bars: 1 nm. The orange, blue, green, red, black and pink circles indicate the points where the differential conductance spectra have been acquired. c) dI/dV spectra acquired at the positions depicted by the colored circles in (b). The reference spectrum taken on the bare Au(111) surface is shown in pink. d) Table summarizing the HOMO and LUMO energy positions determined from the differential conductance spectra.

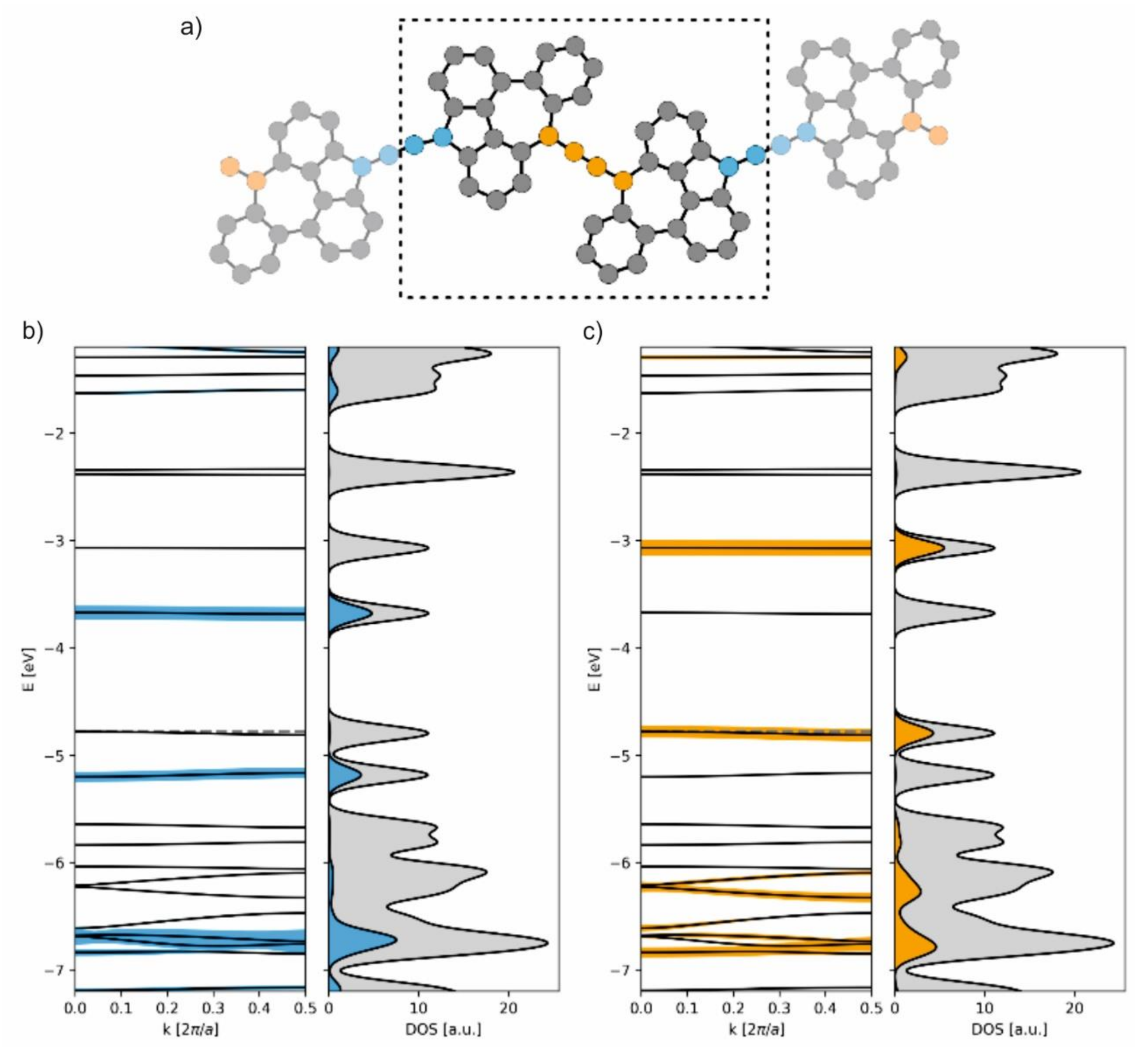

Figure S7. Band structure of the polymer **3** with alternating pentagon-pentagon and heptagon-heptagon cumulene connections. a) Sketch of the geometry of the unit cell. Hydrogens are omitted; circles denote carbon atoms, with blue and orange circle denoting carbon atoms that define pentagon-pentagon and heptagon-heptagon cumulene connections. b) Band structure and the density of states (DOS) of the polymer. The width of the blue line around the bands shows the relative projection of the electronic states on the atoms defining the pentagon-pentagon connection and the blue filled curve on the DOS plots shows the corresponding projected density of states (PDOS). c) Same bands and DOS as b) but with the projection done on the heptagon-heptagon connection (orange). In the band structures, the dashed line shows the valence band onset. The DOS was produced with a Gaussian-broadening with 0.1 eV full width at half maximum.

3. Mass spectra

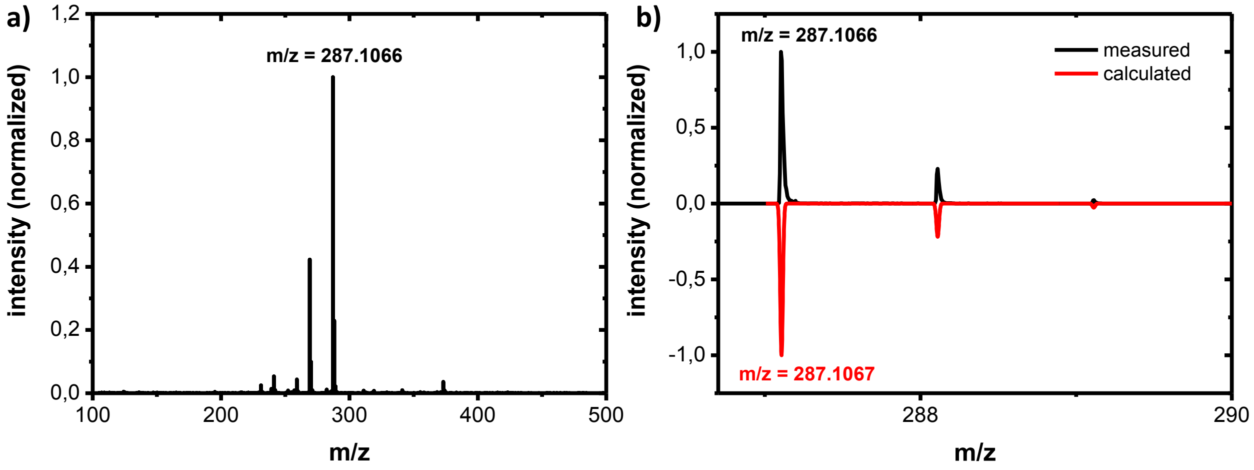

Figure S7. a) HR-APCI-TOF spectrum of **4**; b) HR-APCI-TOF magnified spectrum of **4** (black line) is in agreement to the expected isotopic distribution pattern (red line).

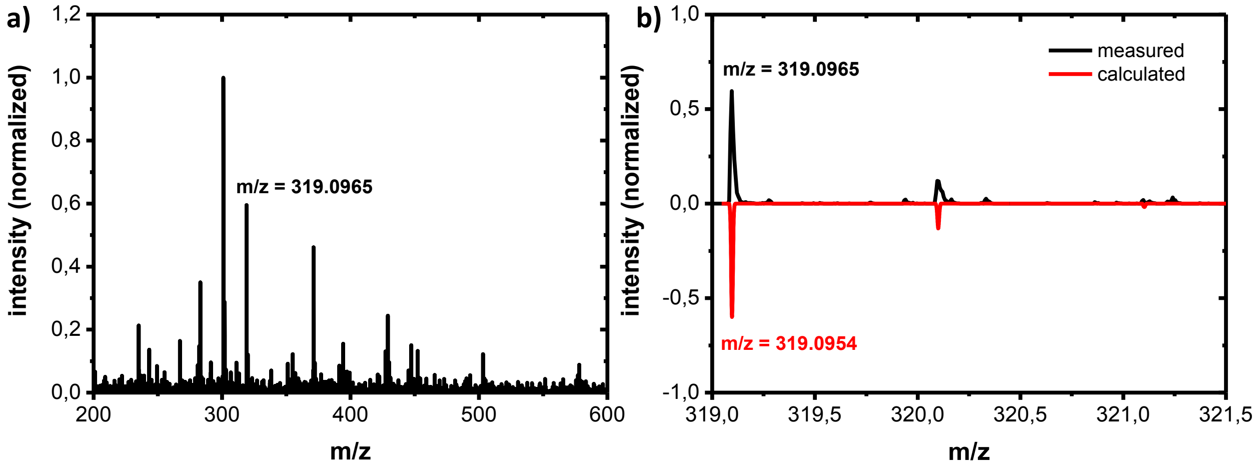

Figure S8. a) HR-APCI-TOF spectrum of **5**; b) HR-APCI-TOF magnified spectrum of **5** (black line) is in agreement to the expected isotopic distribution pattern (red line).

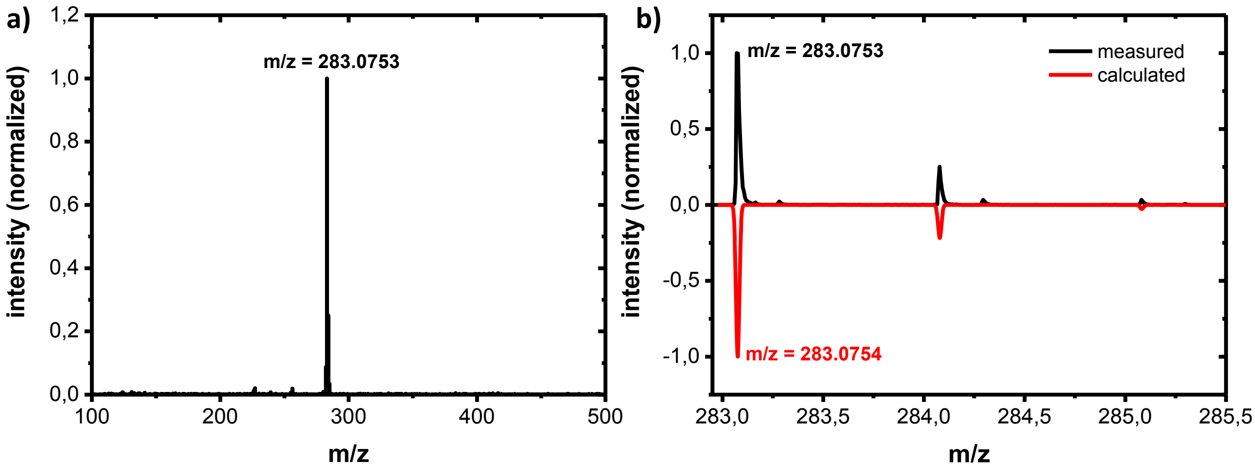

Figure S9. a) HR-APCI-TOF spectrum of **6**; b) HR-APCI-TOF magnified spectrum of **6** (black line) is in agreement to the expected isotopic distribution pattern (red line).

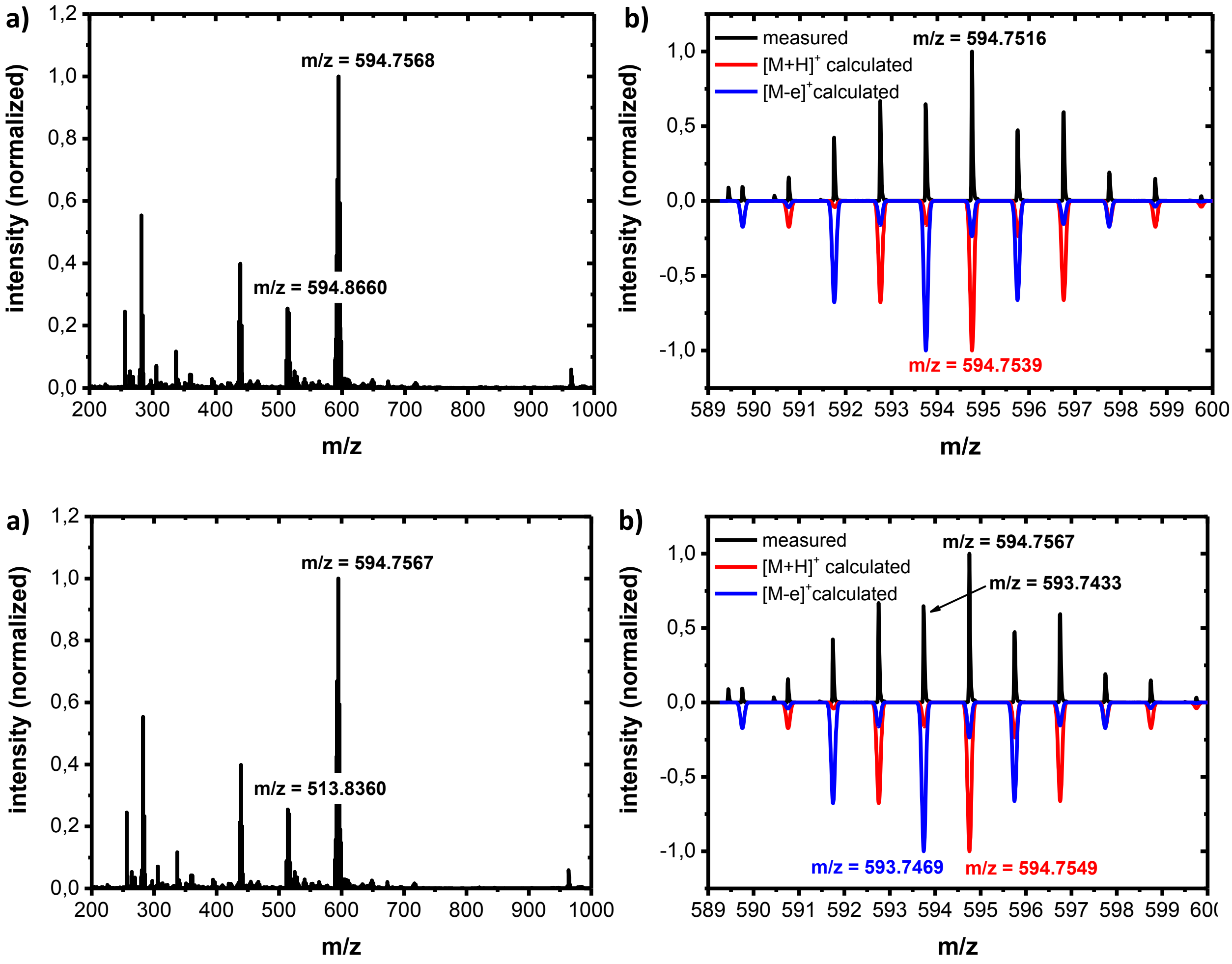

Figure S10. a) HR-APCI-TOF spectrum of **1** ($[M+H]^+$ and $[M\text{-}Br+H]^+$ is marked); b) HR-APCI-TOF magnified spectrum of **1** (black line, superimposition of $[M+H]^+$ and $[M\text{-}e]^+$ species) is in agreement to the expected isotopic distribution pattern (red line for $[M+H]^+$ and blue line for $[M\text{-}e]^+$).

4. NMR spectra

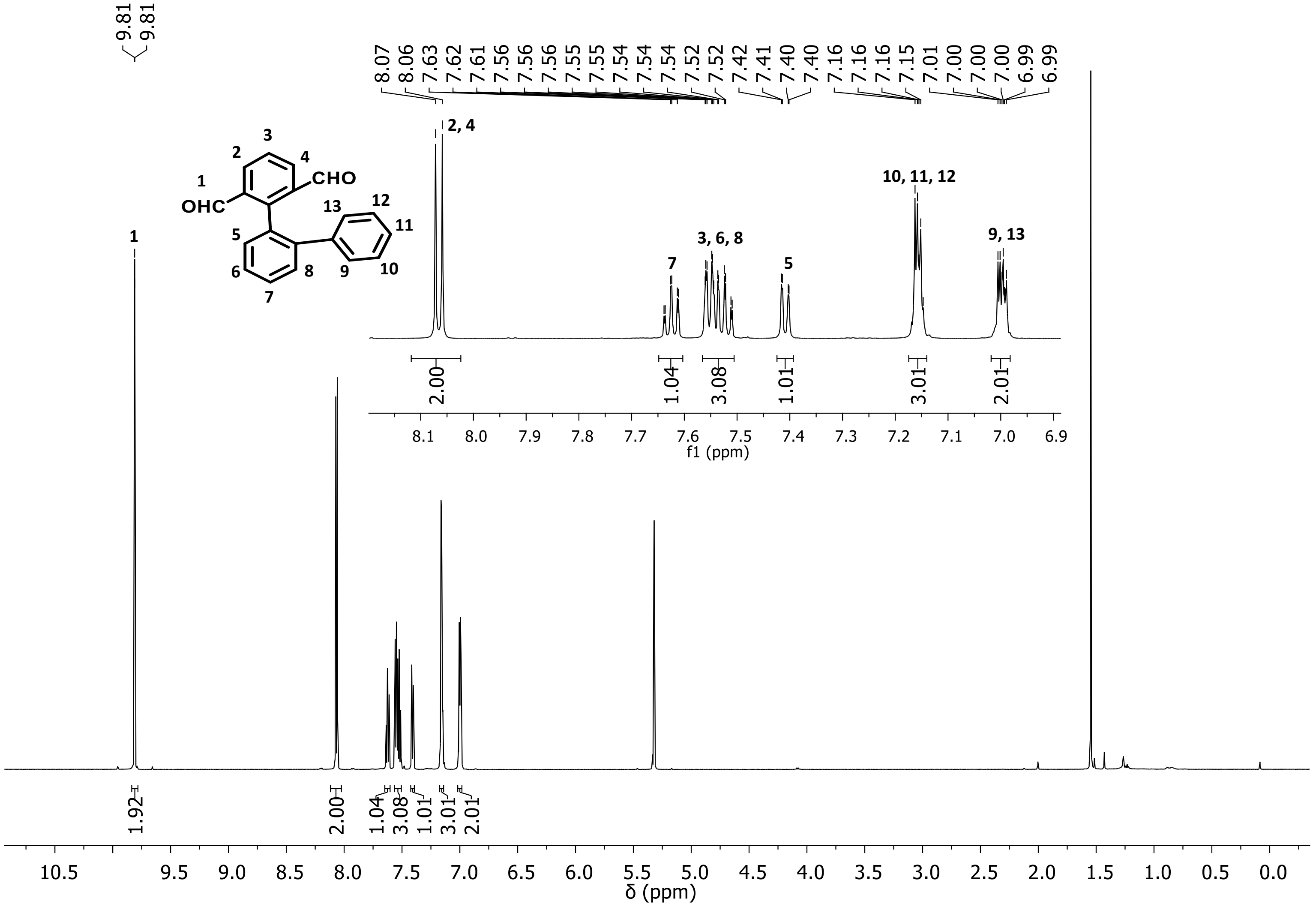

Figure S11. ^{1}H-NMR spectrum of **4** dissolved in dichloromethane-d_2, 600 MHz, 296 K.

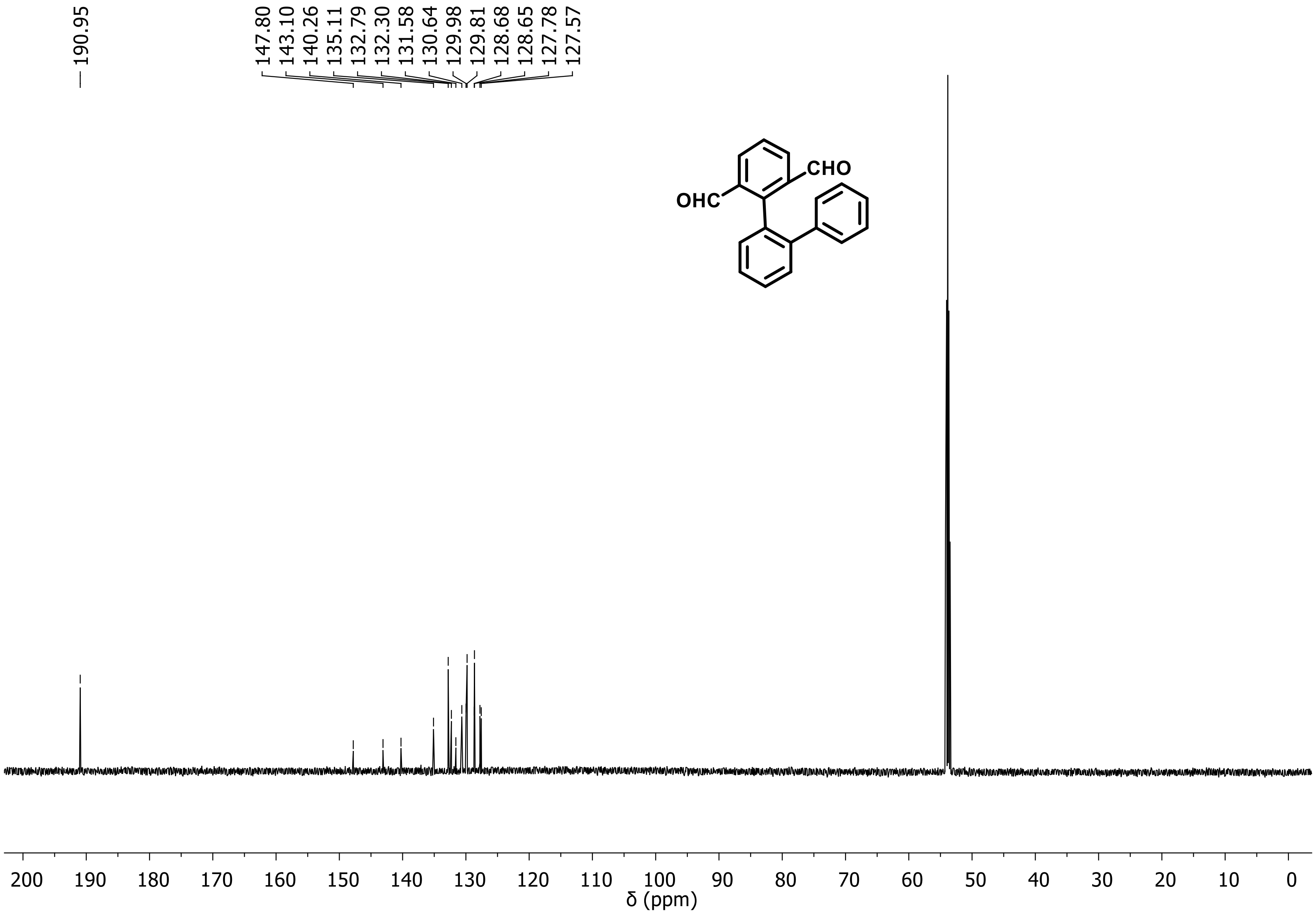

Figure S12. ^{13}C-NMR spectrum of **4** dissolved in dichloromethane -d_2, 150 MHz, 296 K.

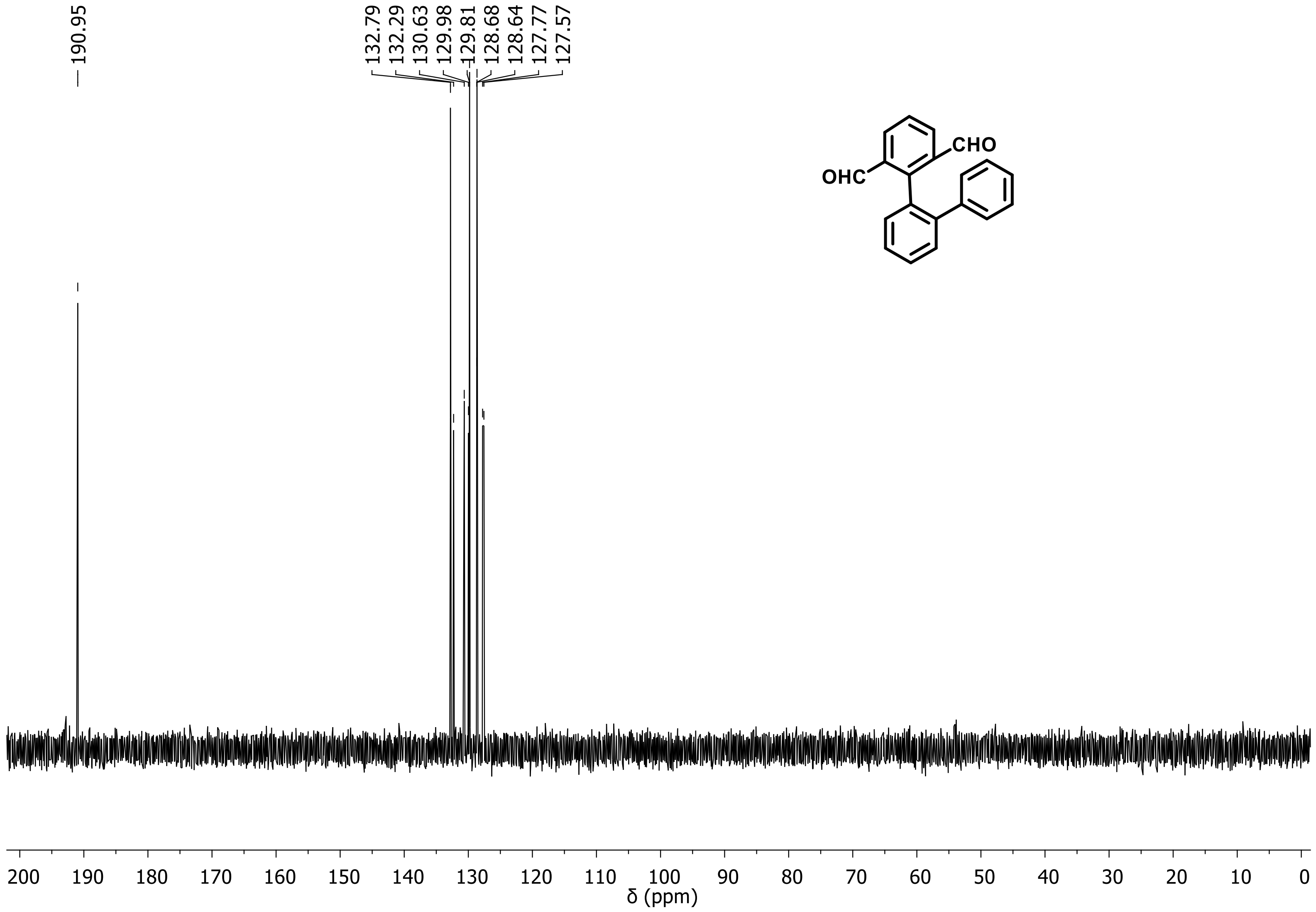

Figure S13. ^{13}C-DEPT135-NMR spectrum of **4** dissolved in dichloromethane -d_2, 150 MHz, 296 K.

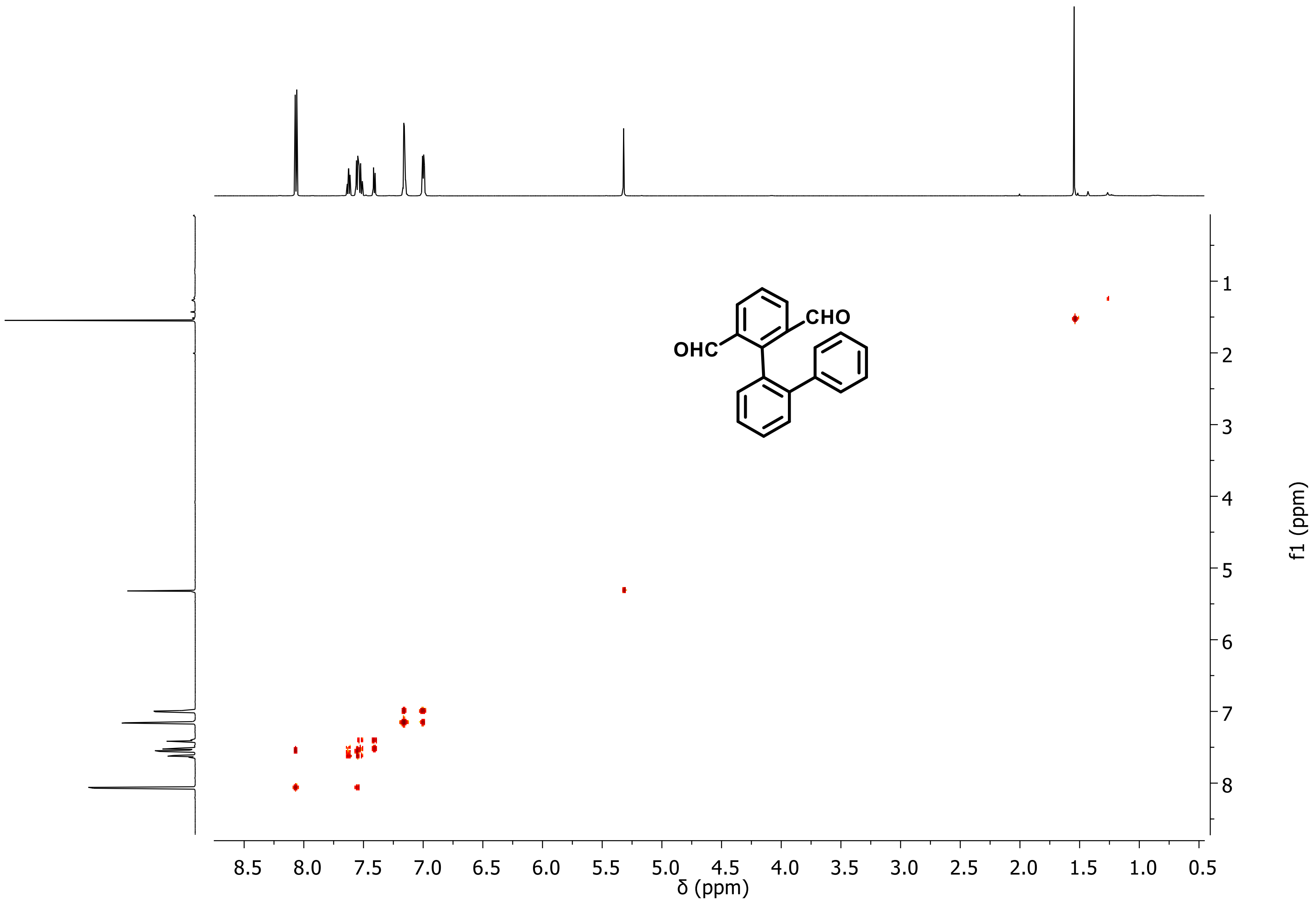

Figure S14. $^1H/^1H$-COSY-NMR spectrum of **4** dissolved in dichloromethane -d_2, 600 MHz, 296 K.

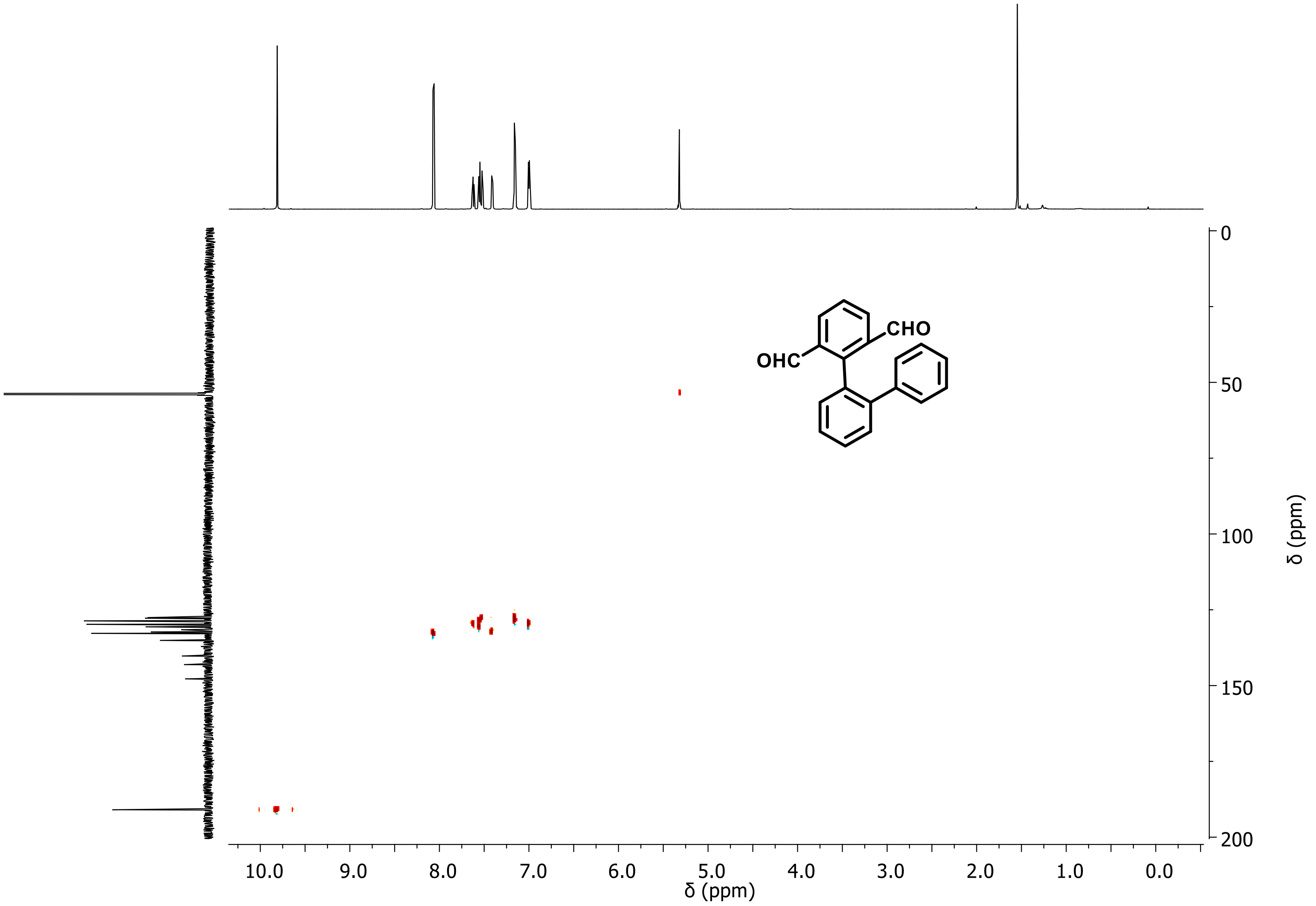

Figure S15. HSQC-NMR spectrum of **4** dissolved in dichloromethane -d2, 150 MHz, 296 K.

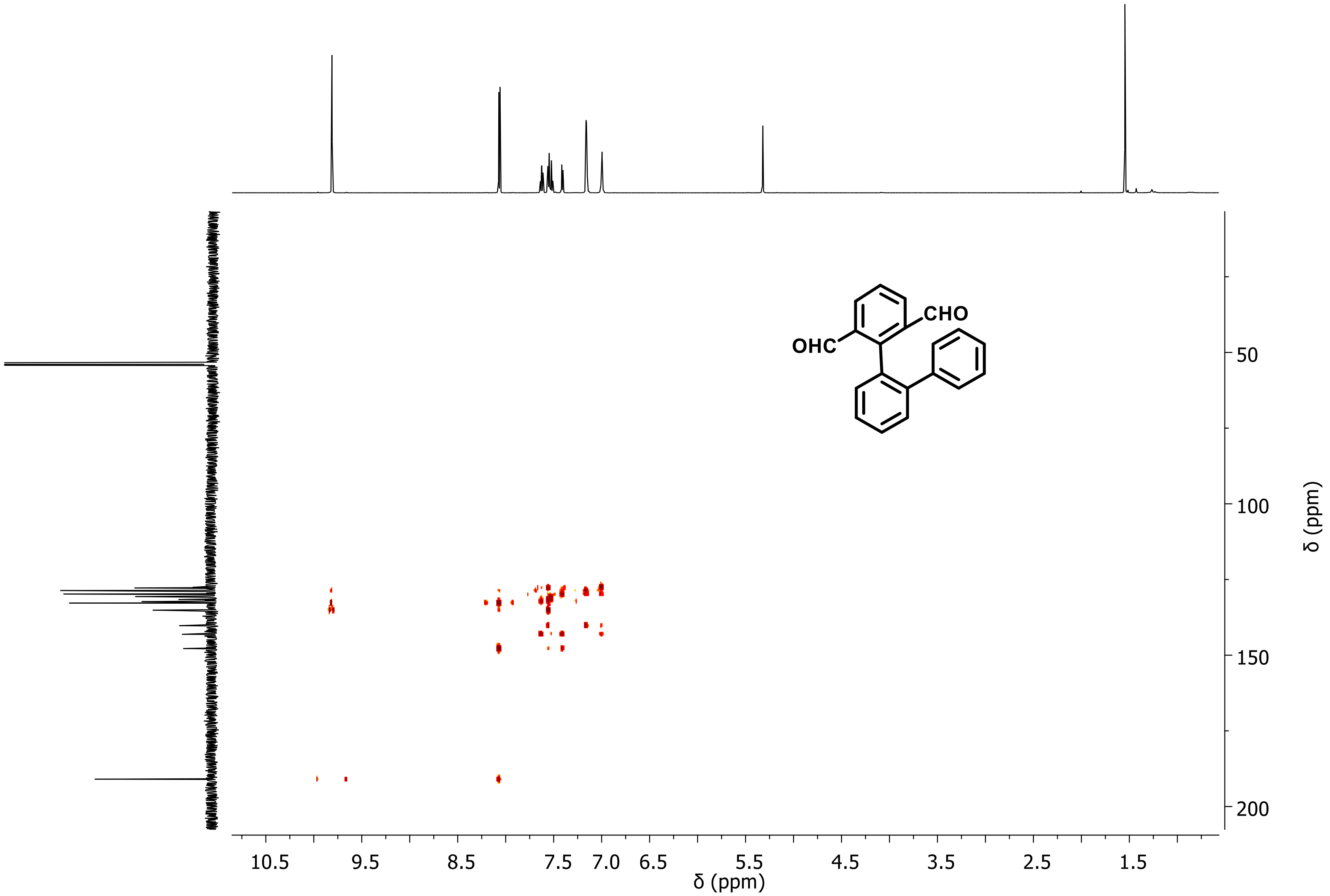

Figure S16. HMBC-NMR spectrum of **4** dissolved in dichloromethane -d2, 150 MHz, 296 K.

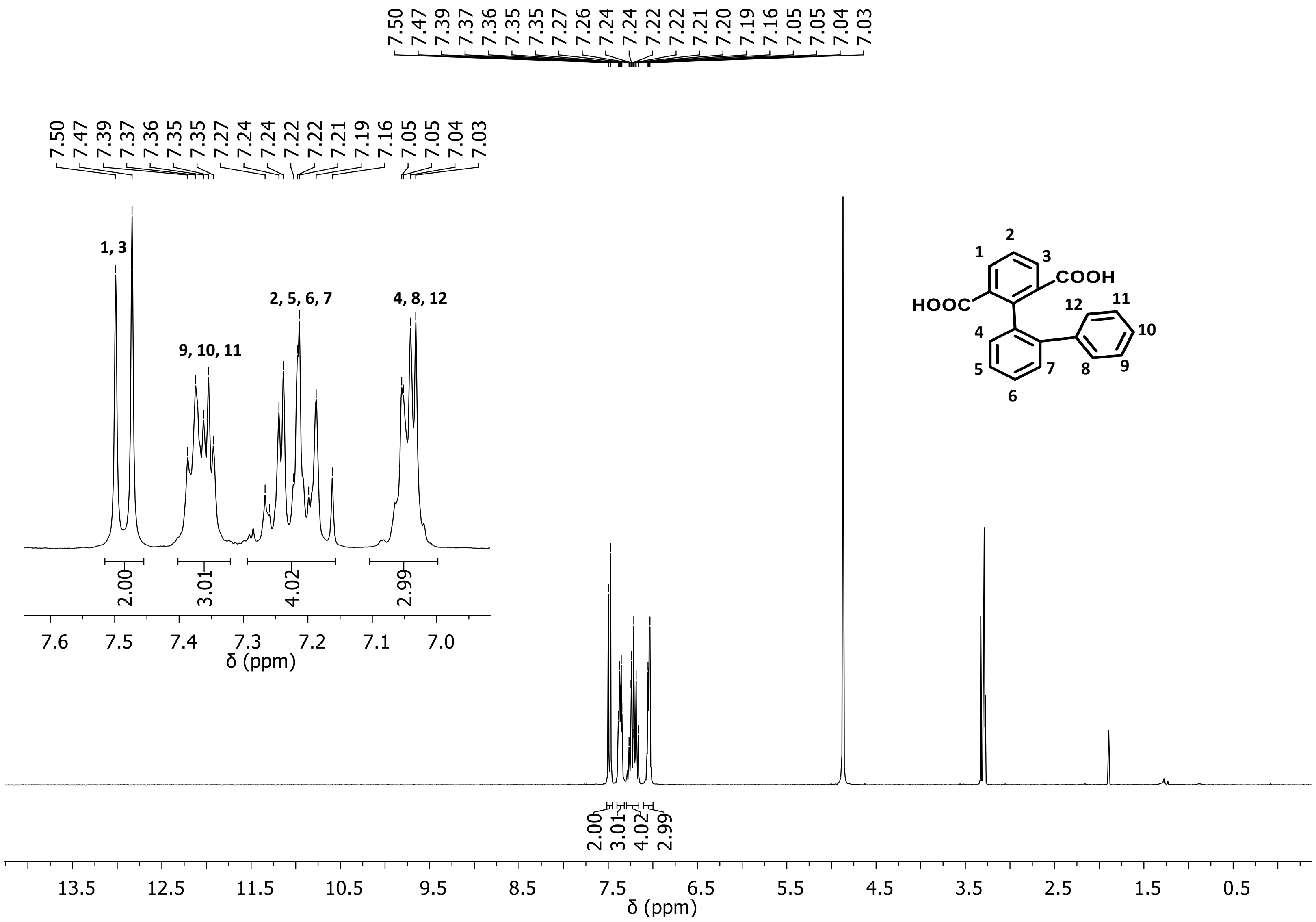

Figure S17. ^{1}H-NMR spectrum of **5** dissolved in methanol-d_4, 300 MHz, 296 K.

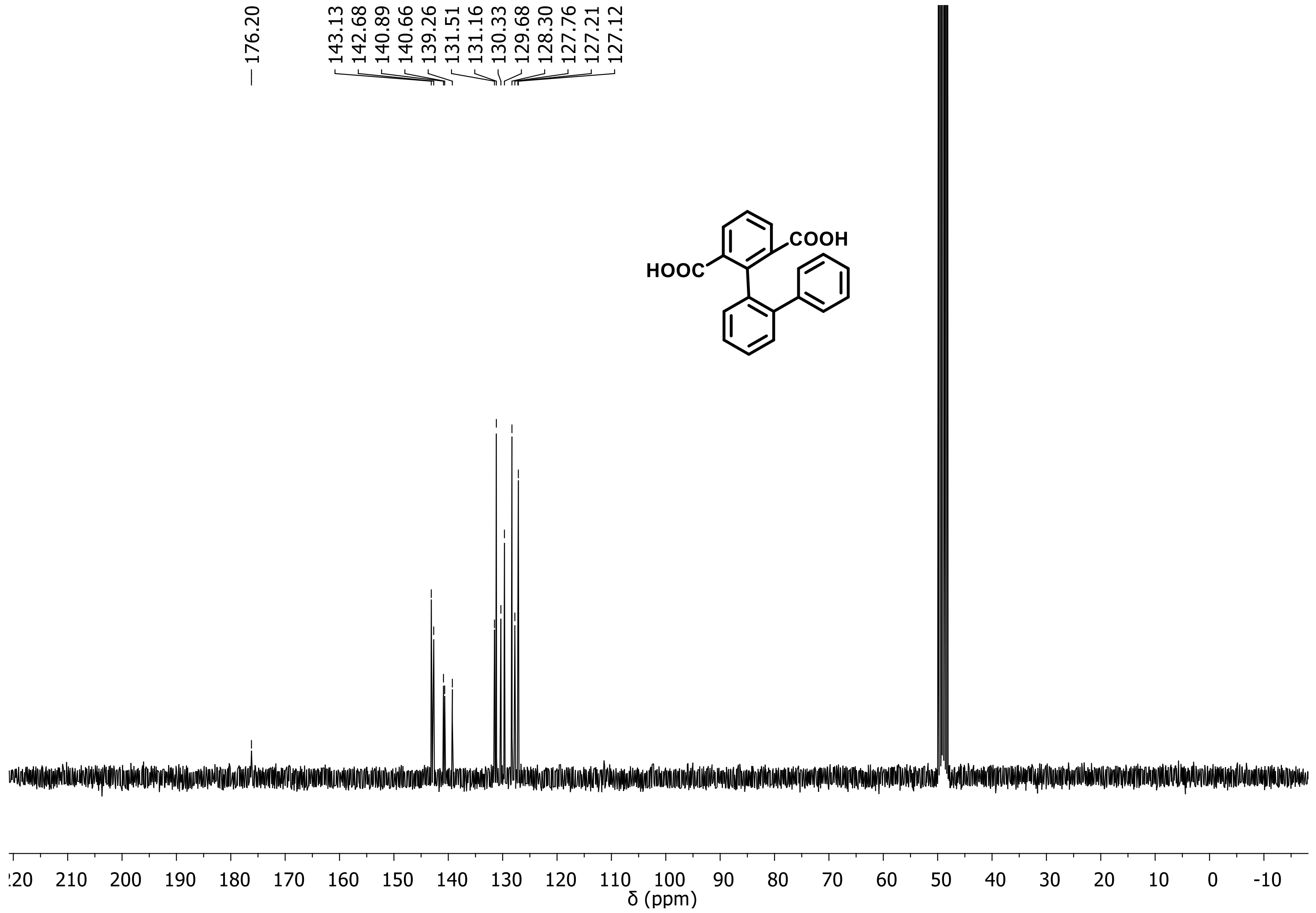

Figure S18. ^{13}C-NMR spectrum of **5** dissolved in methanol-d_4, 75 MHz, 296 K.

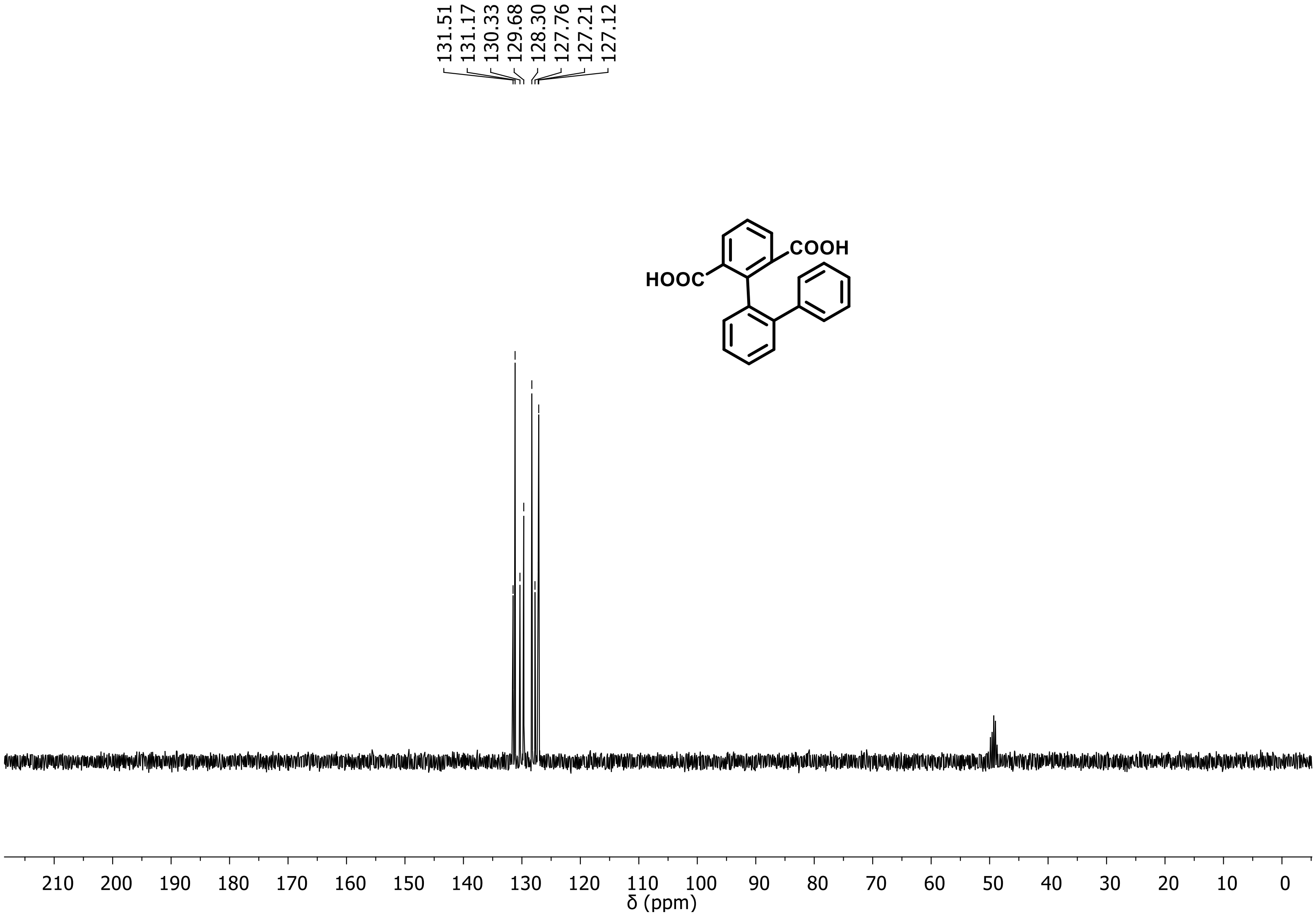

Figure S19. ^{13}C-DEPT135-NMR spectrum of **5** dissolved in methanol-d_4, 75 MHz, 296 K.

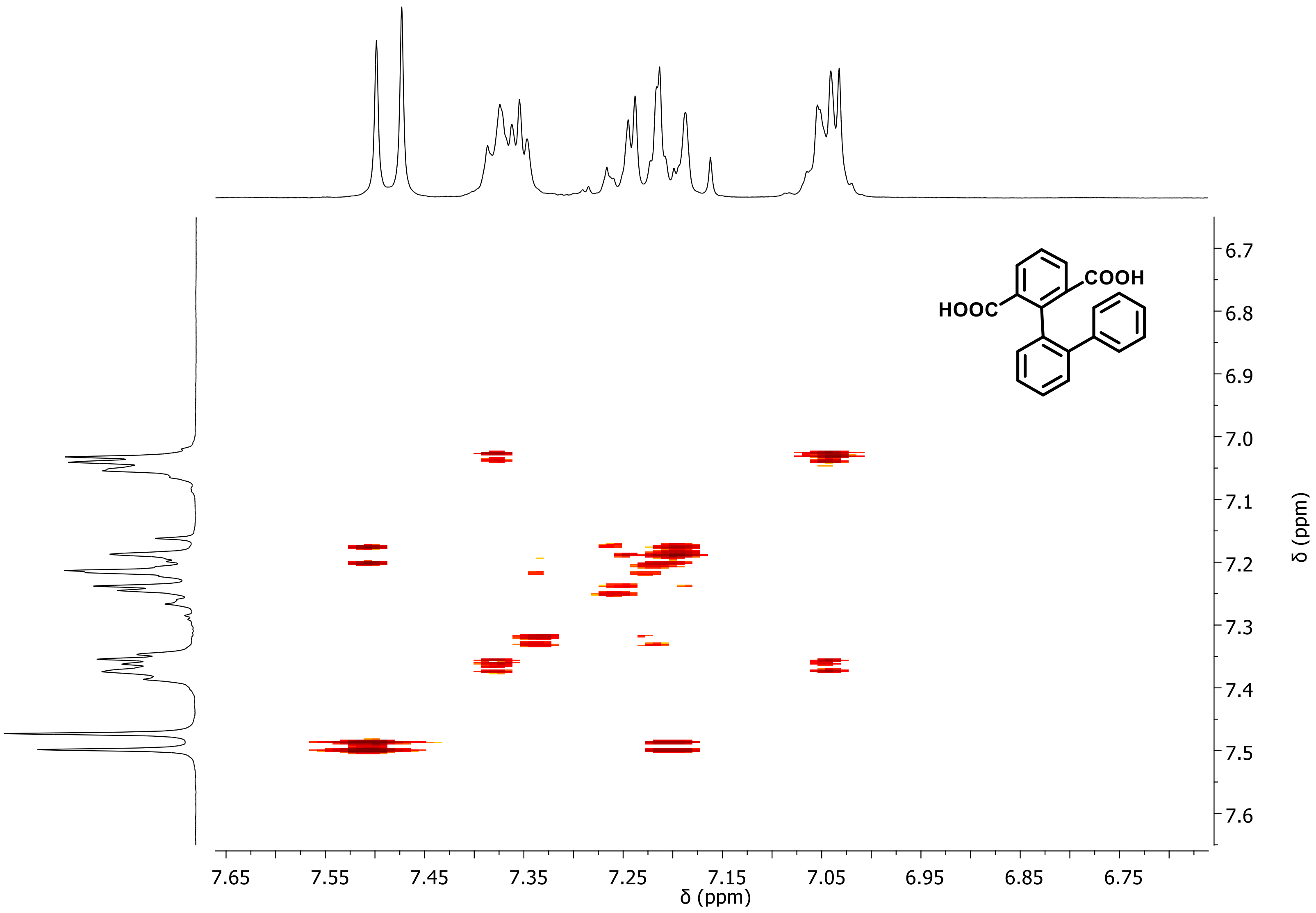

Figure S20. $^1H/^1H$-COSY-NMR spectrum of **5** dissolved in methanol-d_4, 300 MHz, 296 K.

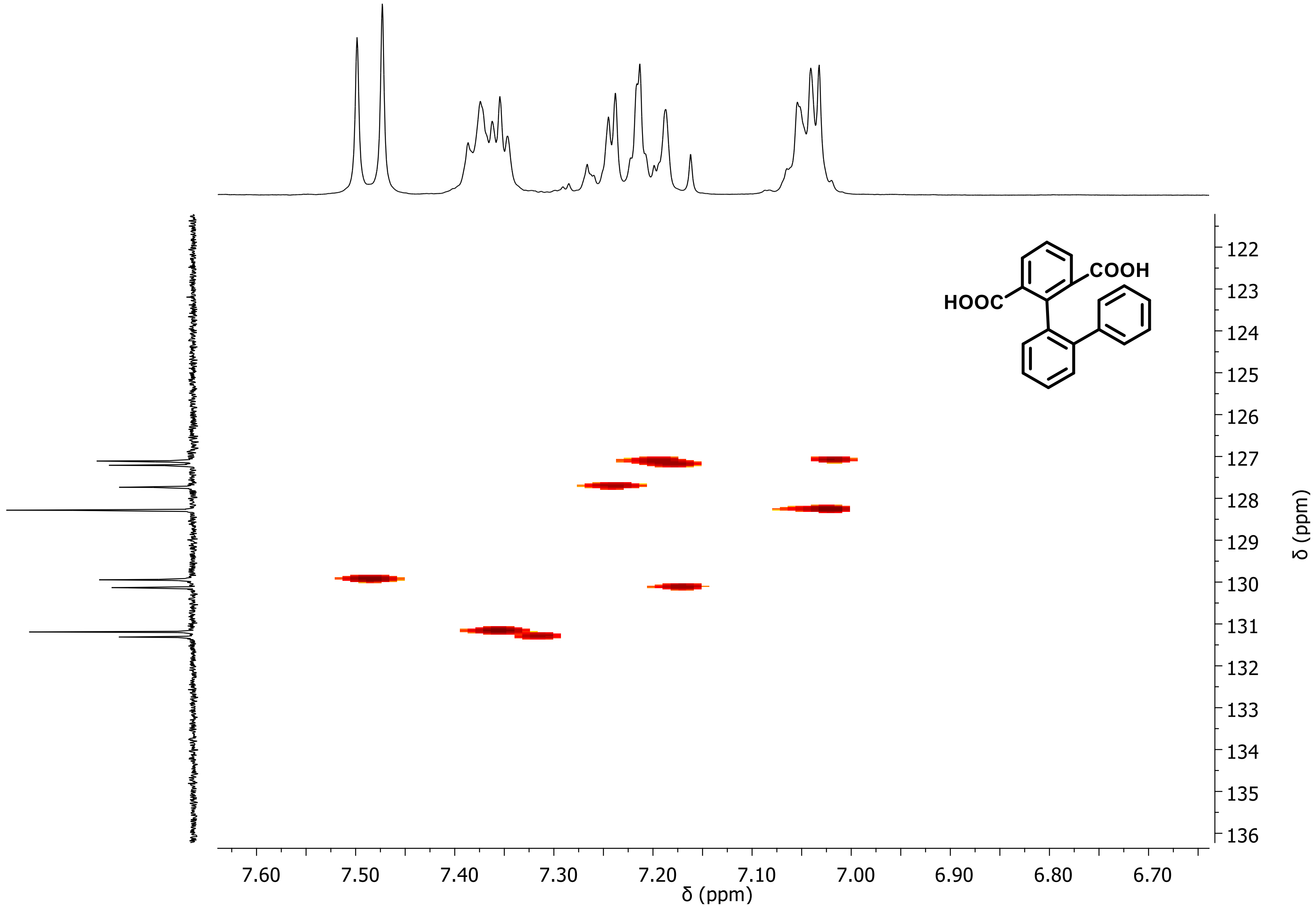

Figure S21. HSQC-NMR spectrum of 5 dissolved in methanol-d_4, 75 MHz, 296 K.

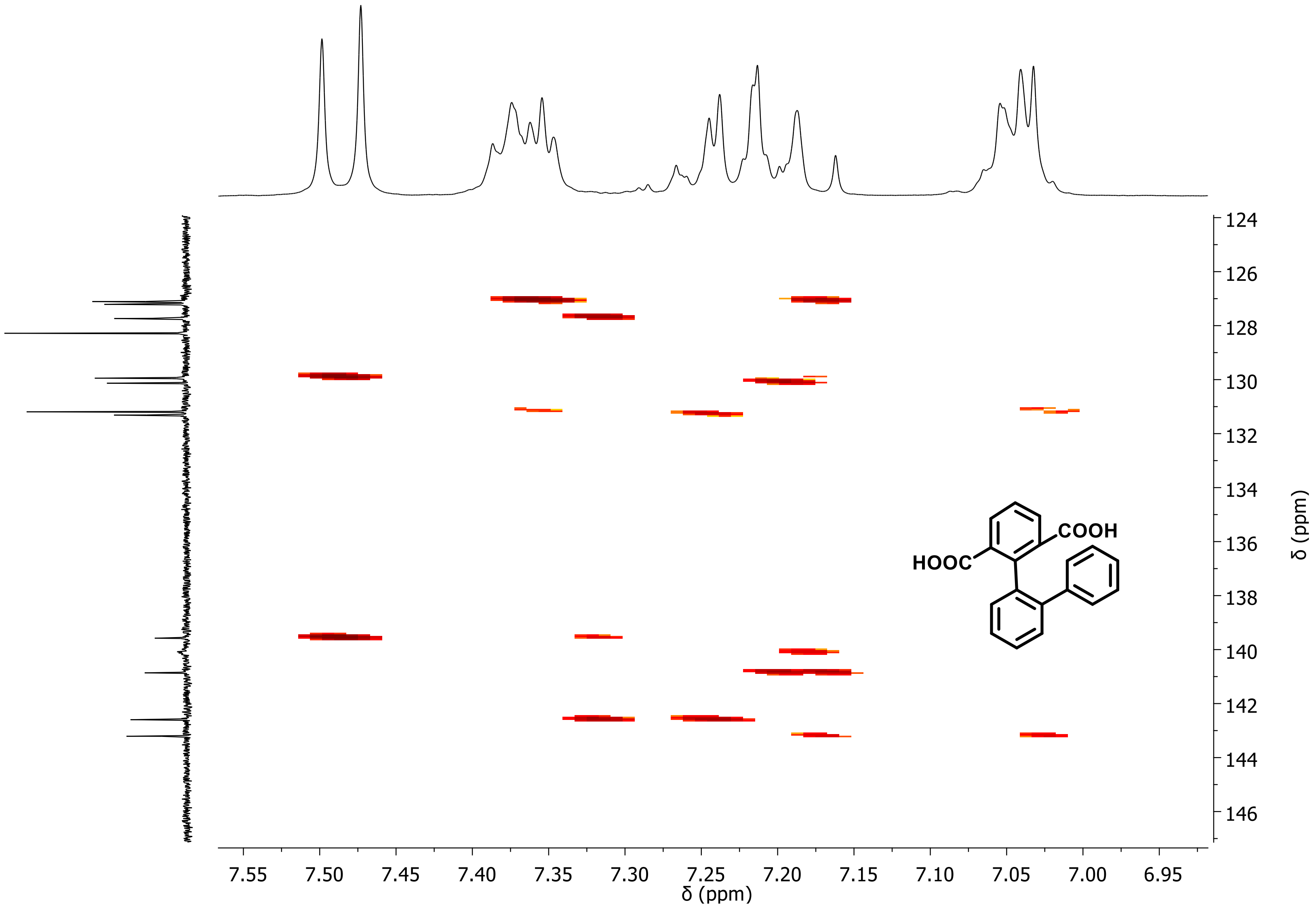

Figure S22. HMBC-NMR spectrum of **5** dissolved in methanol-d4, 75 MHz, 296 K.

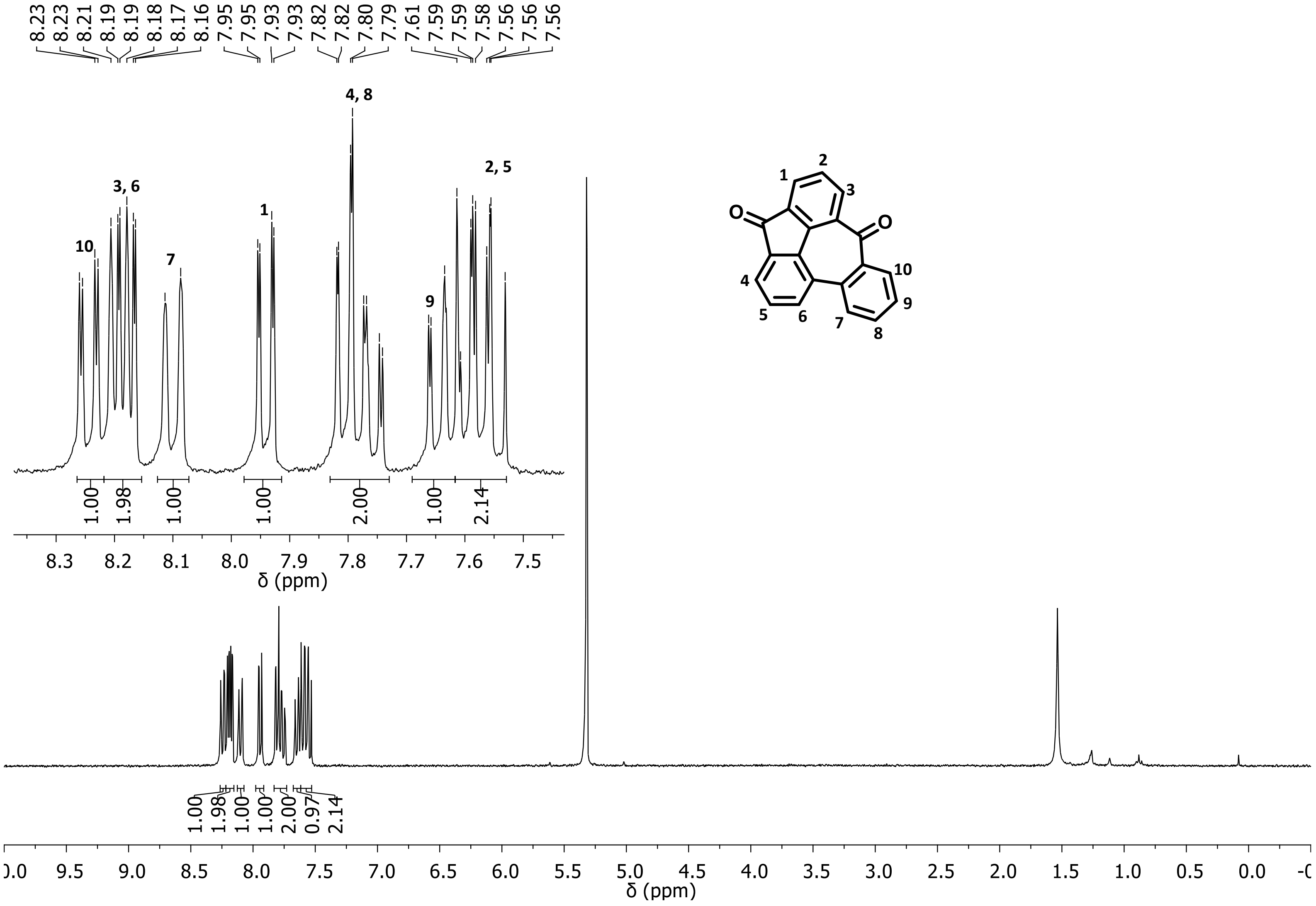

Figure S23. ^{1}H-NMR spectrum of **6** dissolved in dichloromethane-d_2, 300 MHz, 296 K.

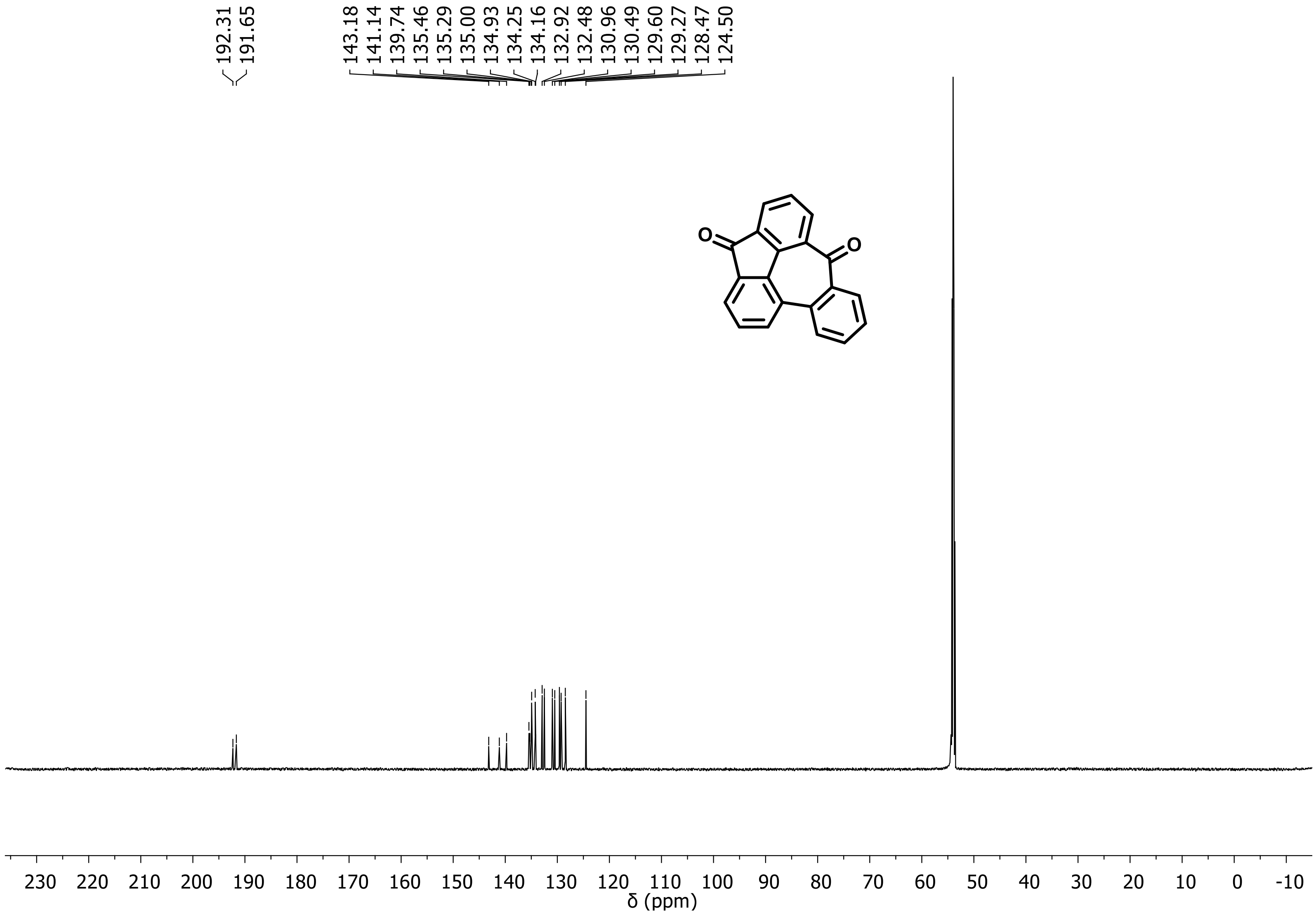

Figure S24. 13C-NMR spectrum of **6** dissolved in dichloromethane-d_2, 150 MHz, 296 K.

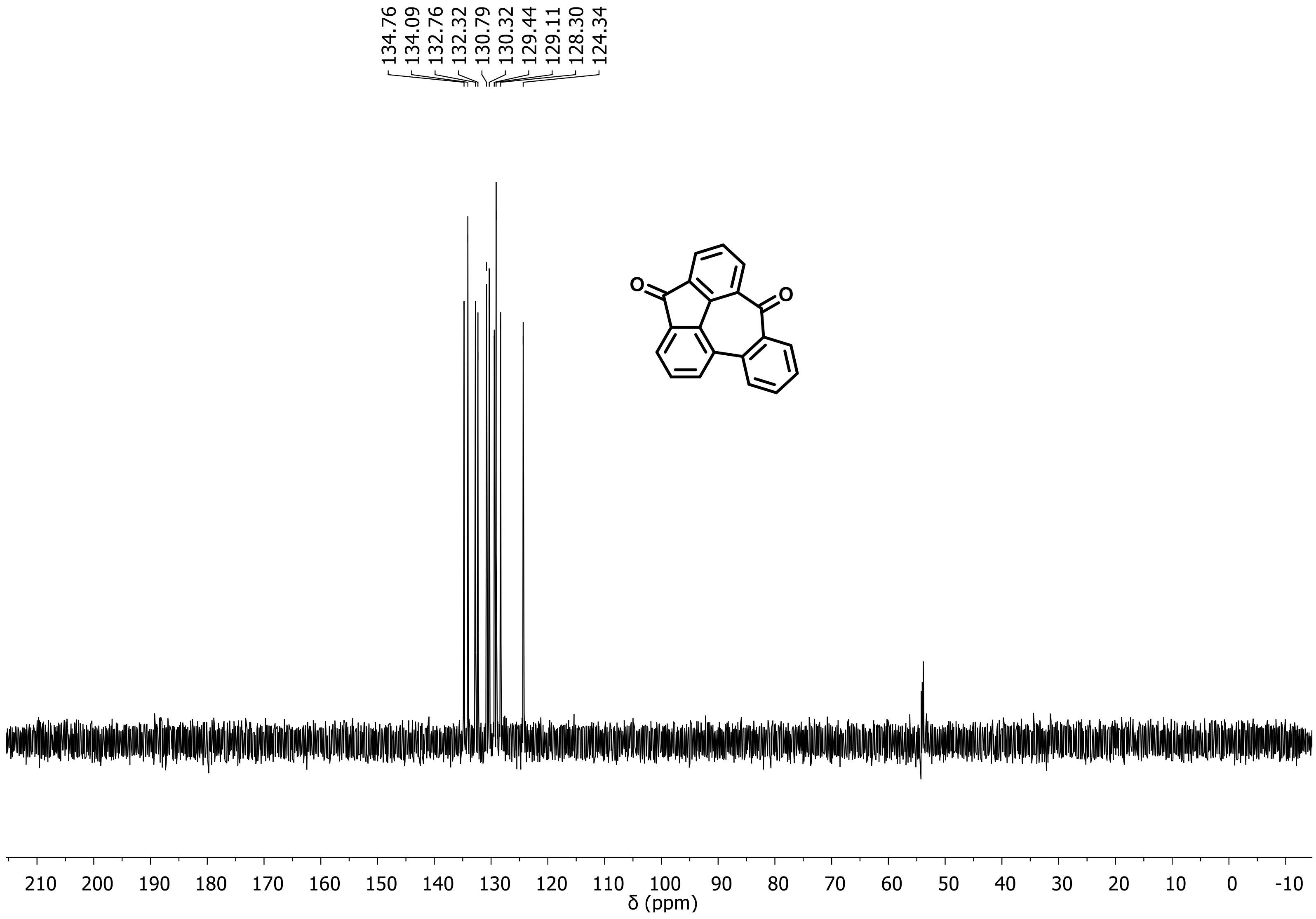

Figure S25. ^{13}C-DEPT135-NMR spectrum of **6** dissolved in dichloromethane-d_2, 150 MHz, 296 K.

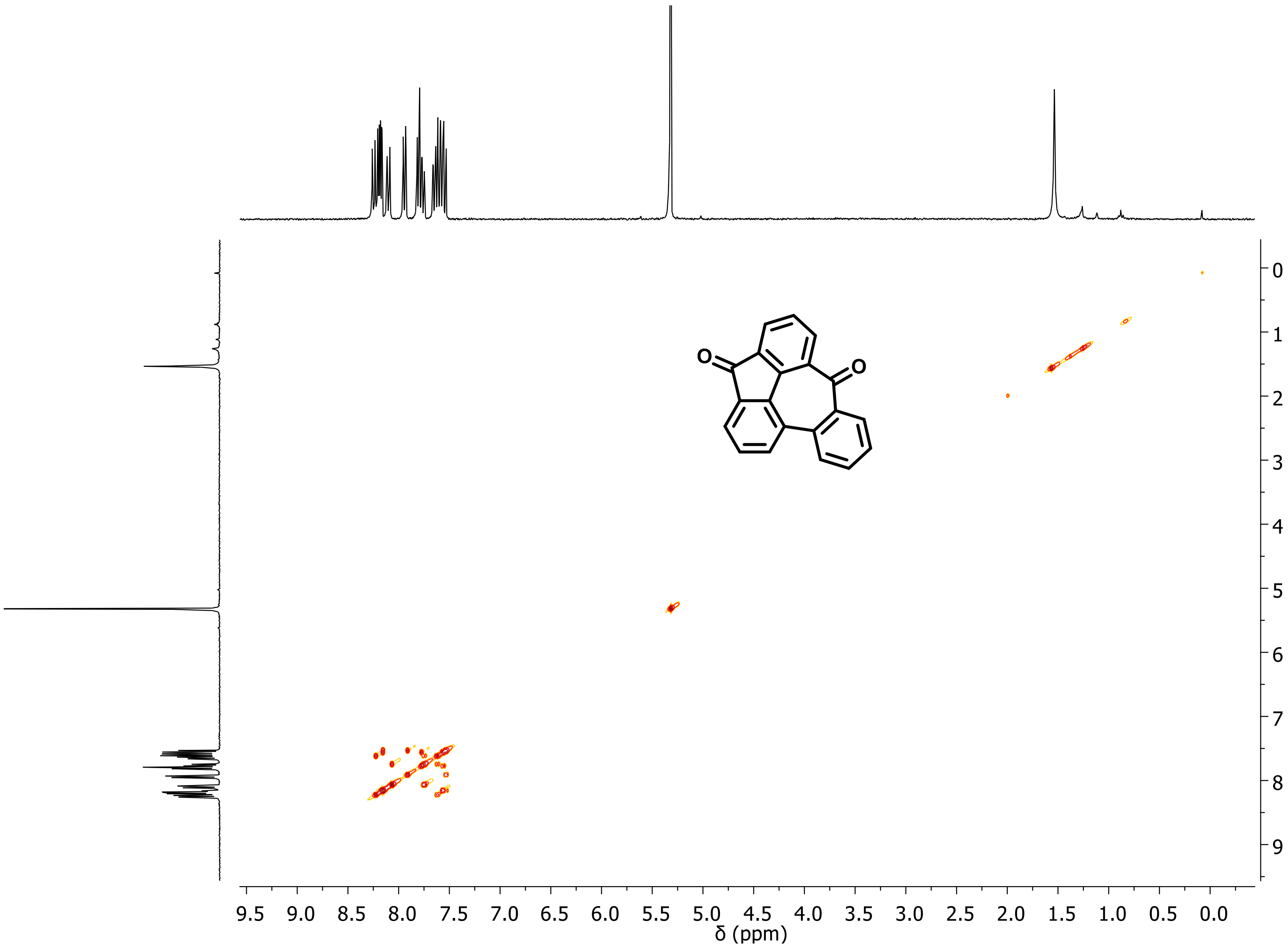

Figure S26. $^1H/^1H$-COSY-NMR spectrum of **6** dissolved in dichloromethane-d_2, 600 MHz, 296 K.

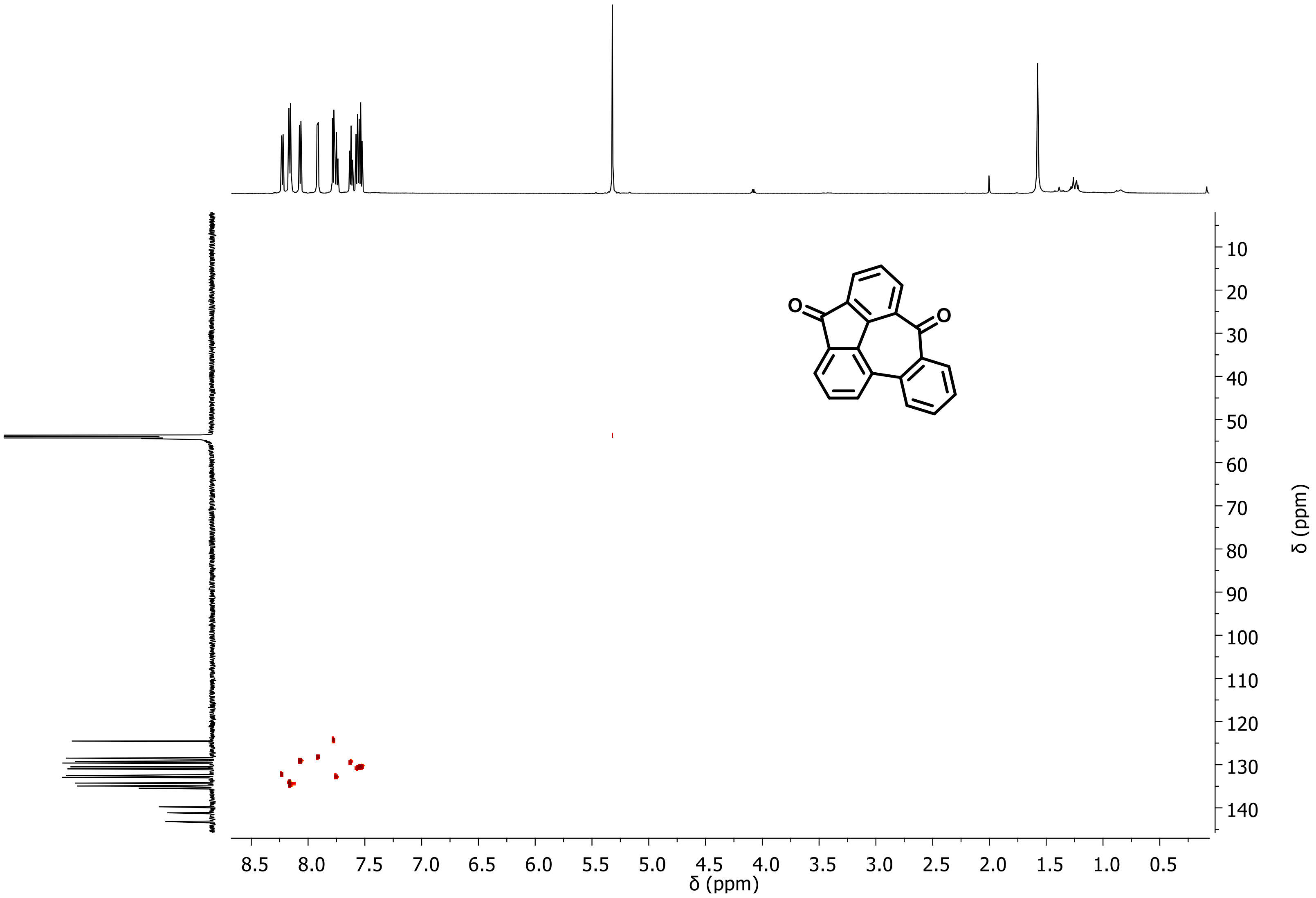

Figure S27. HSQC-NMR spectrum of **6** dissolved in dichloromethane-d_2, 150 MHz, 296 K.

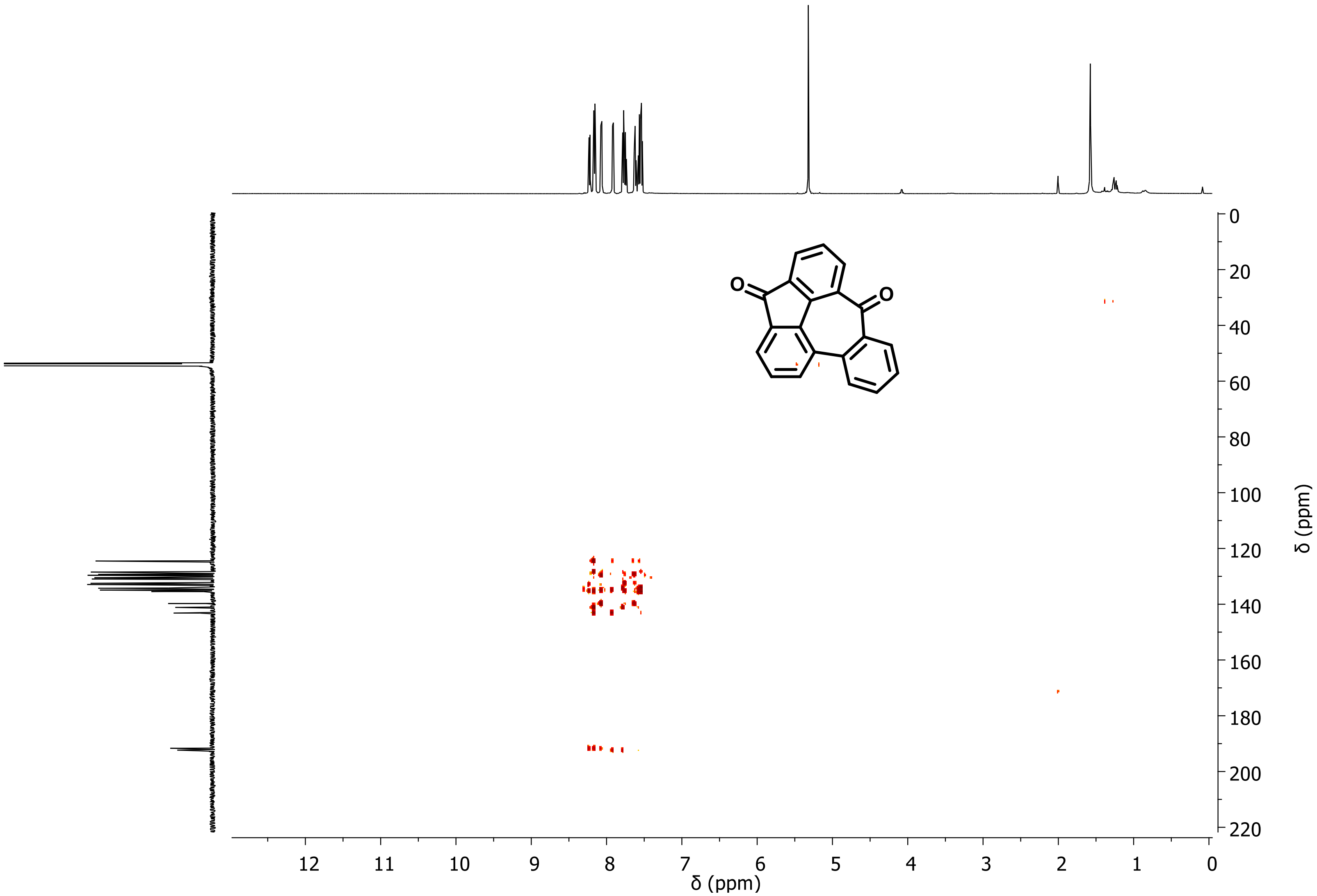

Figure S28. HMBC-NMR spectrum of **6** dissolved in dichloromethane-d_2, 150 MHz, 296 K.

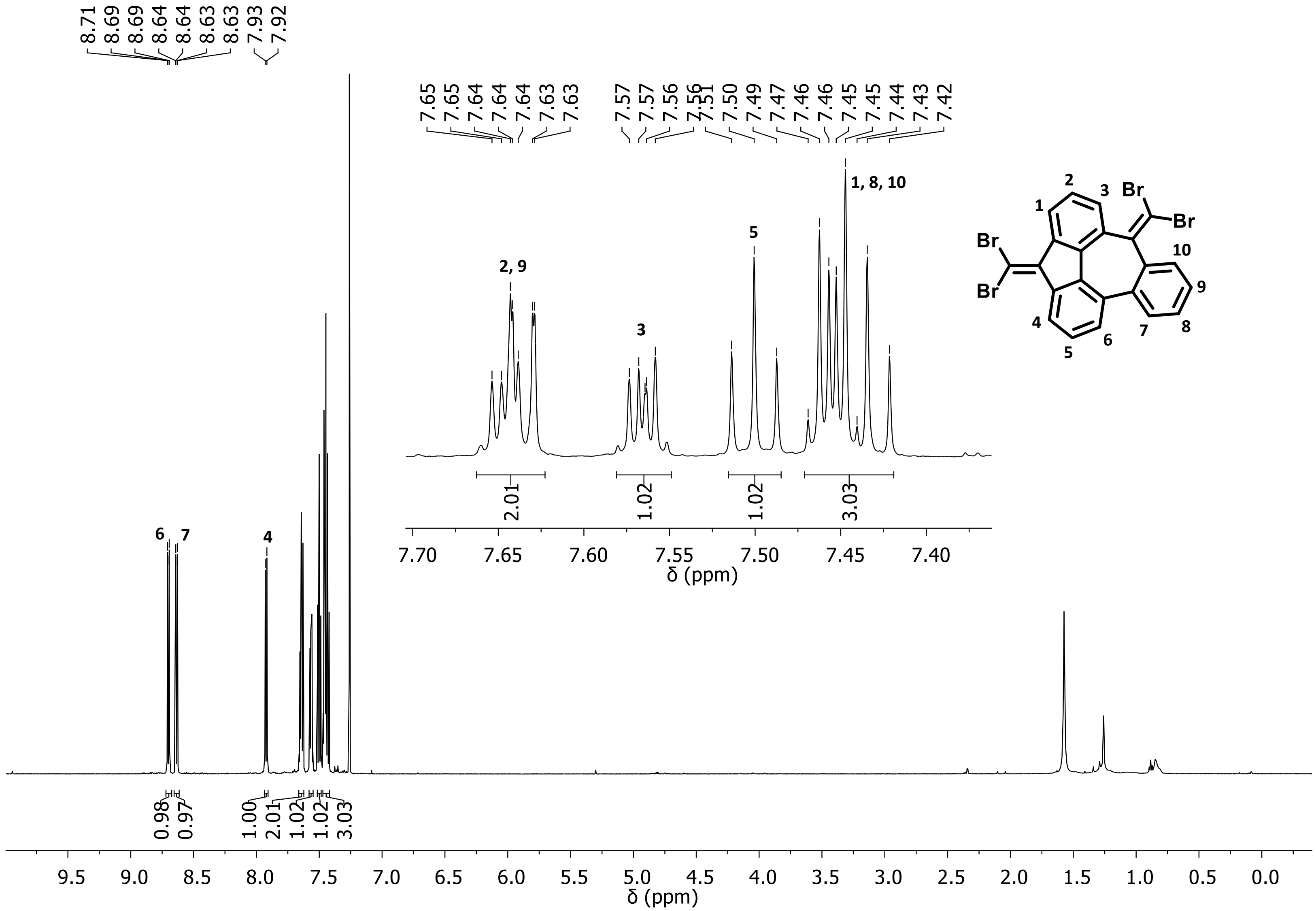

Figure S29. ^{1}H-NMR spectrum of **1** dissolved in chloroform-d, 600 MHz, 296 K.

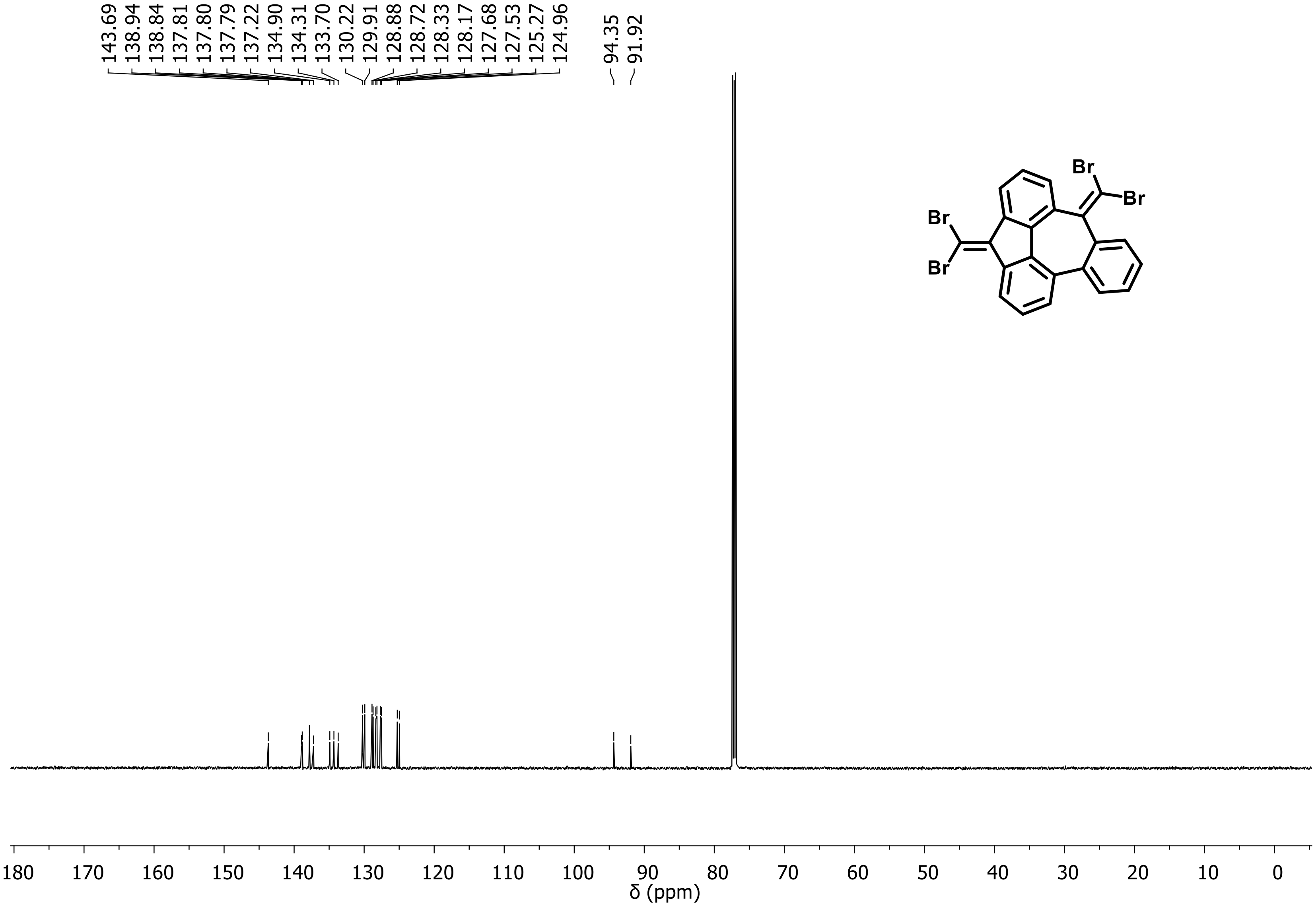

Figure S30. ^{13}C-NMR spectrum of **1** dissolved in chloroform-d, 150 MHz, 296 K.

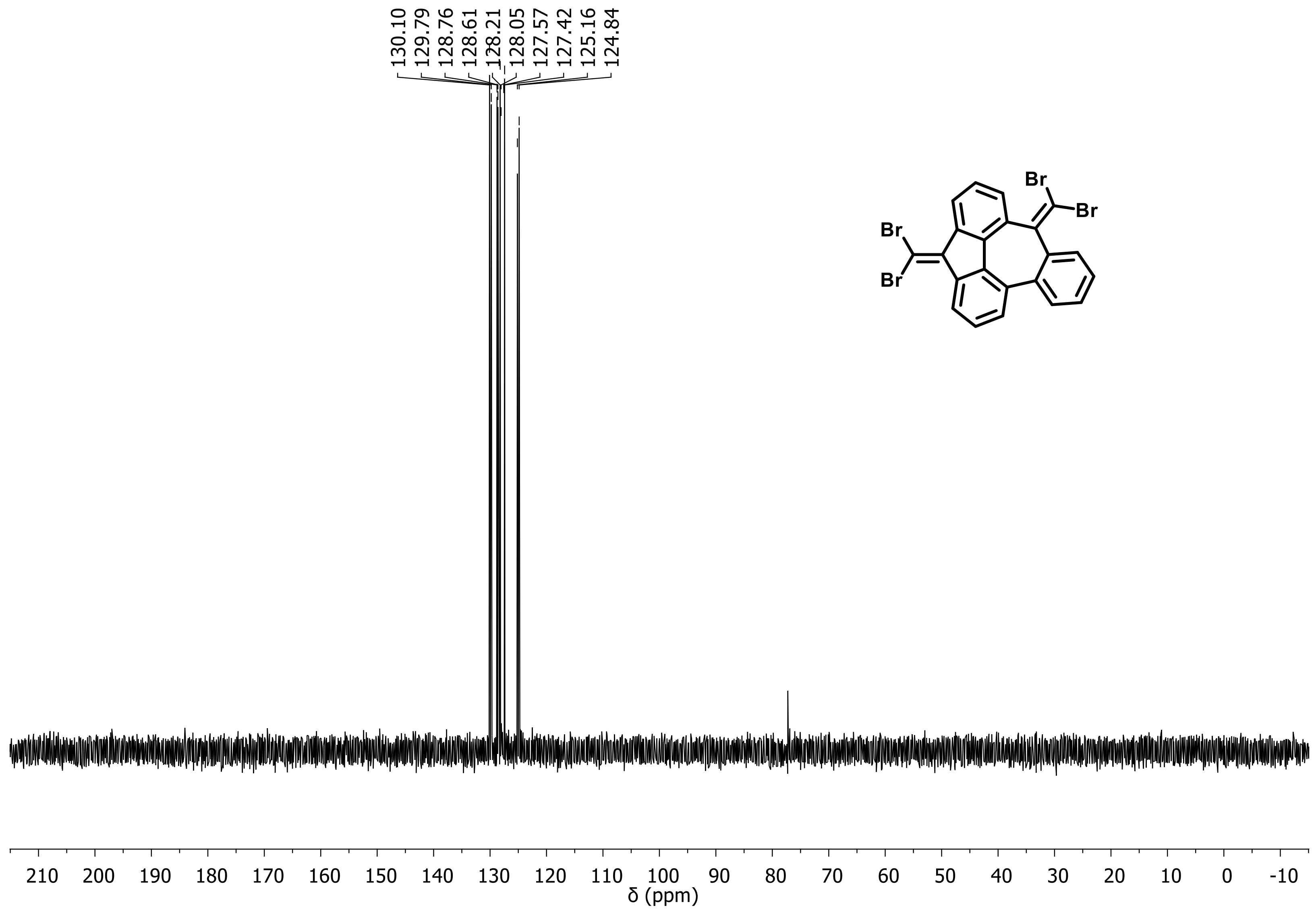

Figure S31. ^{13}C-DEPT135-NMR spectrum of **1** dissolved in chloroform-d, 150 MHz, 296 K.

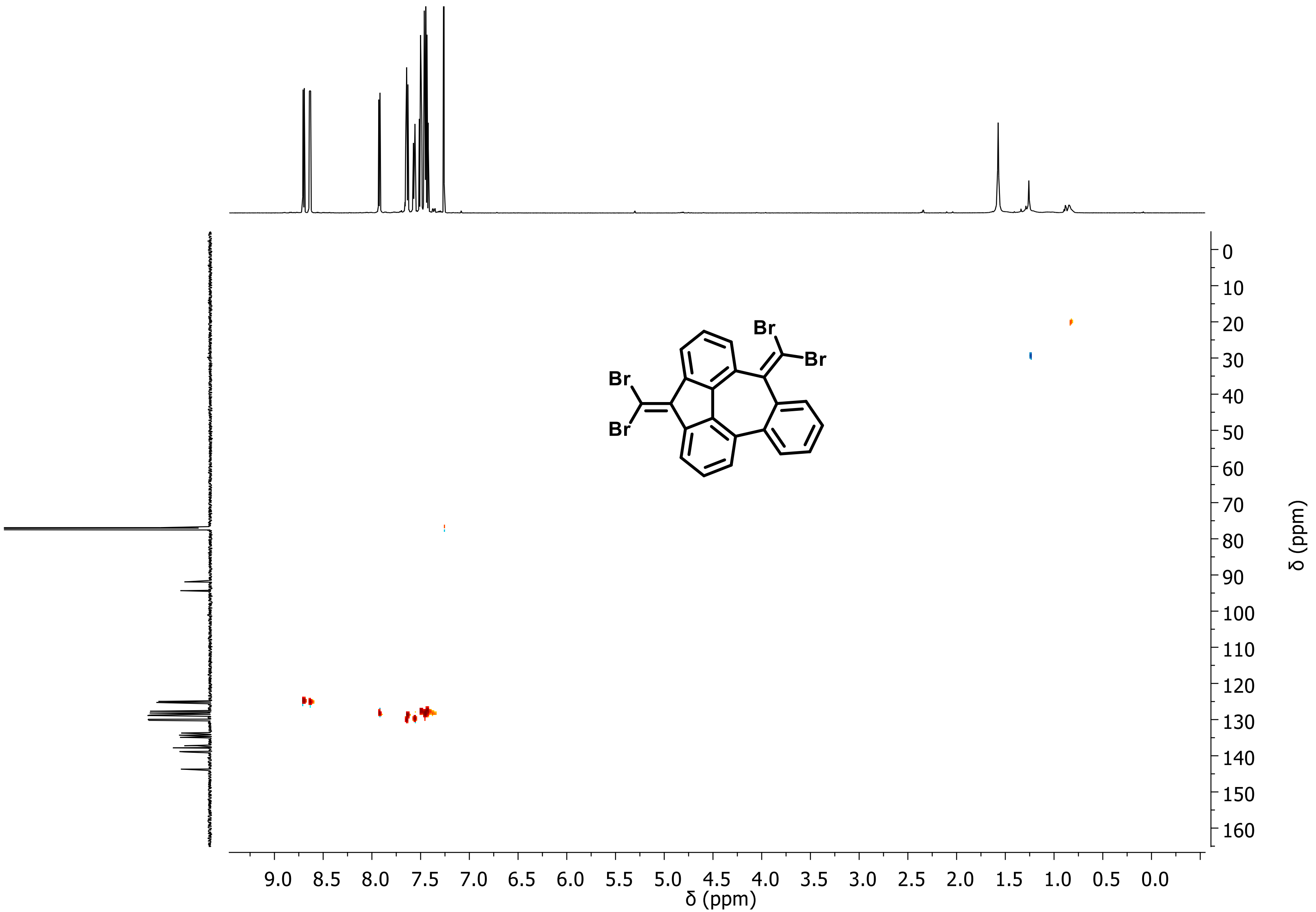

Figure S32. $^{1}H/^{1}H$-COSY-NMR spectrum of **1** dissolved in chloroform-d, 600 MHz, 296 K.

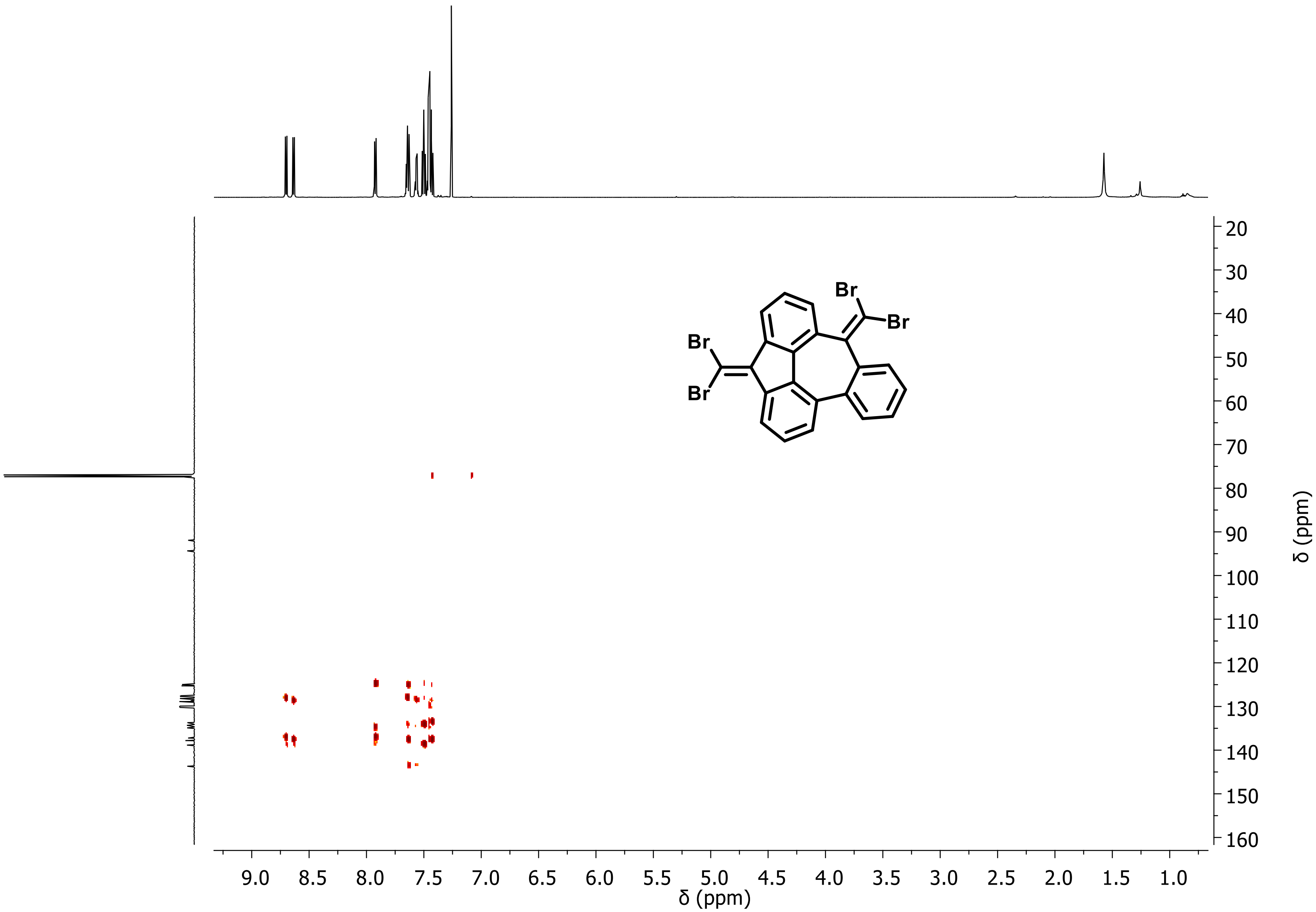

Figure S33. HSQC-NMR spectrum of **1** dissolved in chloroform-d, 150 MHz, 296 K.

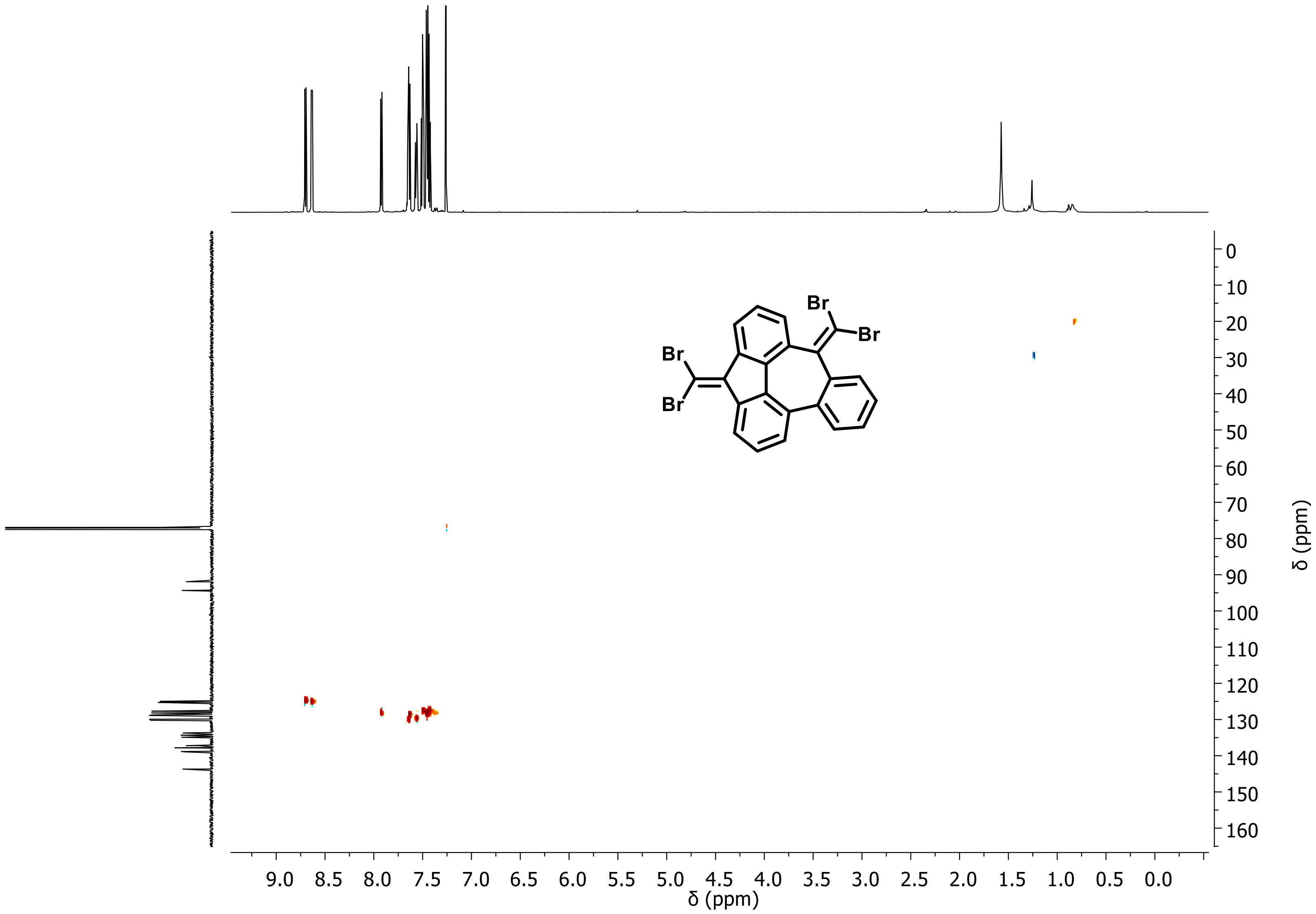

Figure S34. HMBC-NMR spectrum of **1** dissolved in chloroform-d, 150 MHz, 296 K.
